# Stress Hyperglycaemia in Hospitalised Patients and Their 3-Year Risk of Diabetes: A Scottish Retrospective Cohort Study

**DOI:** 10.1371/journal.pmed.1001708

**Published:** 2014-08-19

**Authors:** David A. McAllister, Katherine A. Hughes, Nazir Lone, Nicholas L. Mills, Naveed Sattar, John McKnight, Sarah H. Wild

**Affiliations:** 1 Centre for Population Health Sciences, University of Edinburgh, Edinburgh, United Kingdom; 2 University of Edinburgh/BHF Centre for Cardiovascular Health Science, University of Edinburgh, Edinburgh, United Kingdom; 3 Institute of Cardiovascular and Medical Sciences, BHF Glasgow Cardiovascular Research Centre, University of Glasgow, Glasgow, United Kingdom; 4 Metabolic Unit and Acute Medicine Departments, NHS Lothian, Edinburgh, United Kingdom; Imperial College London, United Kingdom

## Abstract

**Background:**

Hyperglycaemia during hospital admission is common in patients who are not known to have diabetes and is associated with adverse outcomes. The risk of subsequently developing type 2 diabetes, however, is not known.

We linked a national database of hospital admissions with a national register of diabetes to describe the association between admission glucose and the risk of subsequently developing type 2 diabetes.

**Methods and Findings:**

In a retrospective cohort study, patients aged 30 years or older with an emergency admission to hospital between 2004 and 2008 were included. Prevalent and incident diabetes were identified through the Scottish Care Information (SCI)-Diabetes Collaboration national registry. Patients diagnosed prior to or up to 30 days after hospitalisation were defined as prevalent diabetes and were excluded.

The predicted risk of developing incident type 2 diabetes during the 3 years following hospital discharge by admission glucose, age, and sex was obtained from logistic regression models. We performed separate analyses for patients aged 40 and older, and patients aged 30 to 39 years.

Glucose was measured in 86,634 (71.0%) patients aged 40 and older on admission to hospital. The 3-year risk of developing type 2 diabetes was 2.3% (1,952/86,512) overall, was <1% for a glucose ≤5 mmol/l, and increased to approximately 15% at 15 mmol/l. The risks at 7 mmol/l and 11.1 mmol/l were 2.6% (95% CI 2.5–2.7) and 9.9% (95% CI 9.2–10.6), respectively, with one in four (21,828/86,512) and one in 40 (1,798/86,512) patients having glucose levels above each of these cut-points. For patients aged 30–39, the risks at 7 mmol/l and 11.1 mmol/l were 1.0% (95% CI 0.8–1.3) and 7.8% (95% CI 5.7–10.7), respectively, with one in eight (1,588/11,875) and one in 100 (120/11,875) having glucose levels above each of these cut-points.

The risk of diabetes was also associated with age, sex, and socio-economic deprivation, but not with specialty (medical versus surgical), raised white cell count, or co-morbidity. Similar results were obtained for pre-specified sub-groups admitted with myocardial infarction, chronic obstructive pulmonary disease, and stroke.

There were 25,193 deaths (85.8 per 1,000 person-years) over 297,122 person-years, of which 2,406 (8.1 per 1,000 person-years) were attributed to vascular disease. Patients with glucose levels of 11.1 to 15 mmol/l and >15 mmol/l had higher mortality than patients with a glucose of <6.1 mmol/l (hazard ratio 1.54; 95% CI 1.42–1.68 and 2.50; 95% CI 2.14–2.95, respectively) in models adjusting for age and sex.

Limitations of our study include that we did not have data on ethnicity or body mass index, which may have improved prediction and the results have not been validated in non-white populations or populations outside of Scotland.

**Conclusion:**

Plasma glucose measured during an emergency hospital admission predicts subsequent risk of developing type 2 diabetes. Mortality was also 1.5-fold higher in patients with elevated glucose levels. Our findings can be used to inform patients of their long-term risk of type 2 diabetes, and to target lifestyle advice to those patients at highest risk.

*Please see later in the article for the Editors' Summary*

## Abstract 2

In a retrospective analysis of a national database of hospital admissions, David McAllister and colleagues identify the 3-year risk of diabetes of hospitalized patients with hyperglycemia in Scotland.

*Please see later in the article for the Editors' Summary*

## Editors' Summary

**Background:**

Insulin—a hormone released by the pancreas after meals—controls blood glucose (sugar) levels in healthy individuals. However, many patients admitted to hospital because of an acute illness have hyperglycemia, an abnormally high blood glucose level. In this setting, hyperglycemia can be caused by the drugs that patients are taking for existing conditions or may be stress hyperglycemia, a reversible condition in which hormonal changes induced by acute illness stimulate glucose production by the liver. However, hyperglycemia detected during an acute illness may also indicate underlying or incipient type 2 diabetes, a common condition in which blood glucose control fails. Type 2 diabetes can initially be controlled by diet, exercise, and antidiabetic drugs but many patients eventually need insulin injections to control their blood sugar level. Long-term complications of type 2 diabetes, which include an increased risk of heart attacks and stroke, reduce the life expectancy of people with diabetes by about 10 years compared to people without diabetes

**Why Was This Study Done?:**

Prompt diagnosis of type 2 diabetes can minimize its long-term complications, so experts have designed several scoring systems based on lifestyle and other characteristics that allow primary care clinicians to identify the patients who should be tested for diabetes because they are at high risk of developing the condition. Unfortunately, these scoring systems cannot be used to interpret a high blood glucose result obtained during an acute illness so clinicians cannot currently advise their patients on the clinical significance of this type of abnormal glucose reading or make an informed decision about whether follow-up testing is needed. In this retrospective cohort study, the researchers investigate the association between blood glucose levels measured during emergency hospital admissions in Scotland and the risk of developing type 2 diabetes by linking together national databases of hospital admissions, laboratory test results, and people with diabetes. A retrospective cohort study examines the medical histories of a group of patients.

**What Did the Researchers Do and Find?:**

The researchers used the databases to identify more than 100,000 patients aged 30 years or older who were admitted to a hospital for an acute illness between 2004 and 2008 in Scotland, to obtain information on blood glucose levels on admission for nearly three-quarters of these patients, and to identify which patients subsequently developed diabetes. They then used statistical models to estimate the patients' risk of developing type 2 diabetes during the 3 years following hospital discharge. Among patients aged 40 years or older, the overall 3-year risk of developing diabetes was 2.3%. The risk of developing diabetes increased linearly with increasing blood glucose level at admission. Specifically, the 3-year risks at blood glucose levels of 7 mmol/l and 11.1 mmol/l were 2.6% and 9.9%, respectively; because glucose levels fluctuate according to when an individual last ate, fasting blood glucose levels of 7 mmol/l and non-fasting blood glucose levels of 11.1 mmol/l are used as thresholds for the diagnosis of diabetes. The diabetes risk associated with blood glucose levels on admission among 30–39-year-old patients followed a similar pattern but was less marked. Finally, high glucose levels on admission were associated with increased mortality.

**What Do These Findings Mean?:**

These findings indicate that blood glucose measured during an emergency hospital admission predicts the subsequent risk of type 2 diabetes among patients aged 40 years or older (the analysis specified in the researchers' original protocol). Importantly, however, they also suggest that a high blood glucose reading in these circumstances usually indicates stress hyperglycemia rather than type 2 diabetes. The accuracy and generalizability of these findings may be limited by the lack of data on ethnicity or body mass index (a measure of obesity), both of which affect diabetes risk, and by other aspects of the study design. Nevertheless, given their findings, the researchers recommend that any patient with a blood glucose level above 11.1 mmol/l on hospital admission for an acute illness (one in 40 patients in this study) should be offered follow-up testing. In addition, the researchers constructed a risk calculator using their findings that should help clinicians to inform their patients about their long-term risk of diabetes following hyperglycemia during an acute hospital admission and to target lifestyle advice to those patients at the highest risk of type 2 diabetes.

**Additional Information:**

Please access these websites via the online version of this summary at http://dx.doi.org/10.1371/journal.pmed.1001708.

- The US National Diabetes Information Clearinghouse provides information about diabetes and about diabetes prevention (in English and Spanish)
- The UK National Health Service Choices website provides information about type 2 diabetes and about living with diabetes; it also provides people's stories about diabetes
- The charity Diabetes UK provides information about diabetes in several languages, including information on healthy lifestyles for people with diabetes
- Wikipedia has a page on stress hyperglycemia (note that Wikipedia is a free online encyclopedia that anyone can edit; available in several languages)
- More information about stress hyperglycemia is available in Diapedia, a living textbook of diabetes produced by the European Association for the Study of Diabetes
- GUARD (Glucose on Unselected Admissions and Risk of Diabetes), a risk calculator that allows clinicians to estimate a patient's 3-year risk of diabetes following hyperglycemia at hospital admission for an acute illness, is available online
- The UK-based non-profit organization Healthtalkonline has interviews with people about their experiences of diabetes
- MedlinePlus provides links to further resources and advice about diabetes and diabetes prevention (in English and Spanish)

## Introduction

Hyperglycaemia detected during acute illness is associated with adverse outcomes. Among patients without known diabetes admitted to hospital with myocardial infarction (MI), stroke, pneumonia, and exacerbation of chronic obstructive pulmonary disease (COPD), higher glucose levels are associated with in-hospital and longer-term mortality, intensive care unit admission, prolonged length of stay, and discharge to long-term nursing care [1]–[8].

Hyperglycaemia during acute illness may be caused by drugs such as systemic corticosteroids, thiazides, phenytoin, phenothiazines, protease-inhibitors, and beta-agonists [9]–[11] or as a result of “stress hyperglycaemia” where counter-regulatory hormones such as glucagon, cortisol, catecholamines, and growth hormone promote hepatic gluconeogenesis [12]. Hyperglycaemia detected during acute illness may also be the first clinical evidence of underlying or incipient type 2 diabetes.

In gestational diabetes, an analogous condition wherein hyperglycaemia detected during pregnancy can either be due to pre-existing undiagnosed diabetes or to physiological changes that occur during pregnancy, the risk of persistent hyperglycaemia resulting in a diagnosis of diabetes after delivery has been established in large cohort studies [13]. The same is not true for acute illness related hyperglycaemia, however, as follow-up studies examining this question have generally been limited to specific diseases (coronary disease [14]–[17], stroke [18],[19], or pneumonia [20]) and have generally been small, of short duration, and with considerable loss to follow-up [21]–[28].

A number of scores allow clinicians to select which patients in primary care have a sufficiently high risk of type 2 diabetes to merit a blood glucose test, on the basis of demographic, lifestyle, and anthropometric characteristics [29],[30]. These scores, however, do not help clinicians faced with interpreting an abnormally high result when glucose has already been measured as part of a routine panel of blood tests following admission to hospital, and where it is uncertain whether stress hyperglycaemia or type 2 diabetes is the underlying cause. Consequently, clinicians are currently unable either to advise patients on the clinical significance of raised glucose during acute illness, or to decide what if any follow-up testing is appropriate.

Scottish Care Information (SCI)-Diabetes Collaboration (SCI-DC) is a national register including over 99% of people with diabetes in Scotland [31]. All emergency hospitalisations are recorded on the national Scottish Morbidity Record (SMR01) and all biochemistry and haematology laboratory measurements are recorded in the SCI-Store databases in each Health Board.

Using these large representative routine data sources, we describe the association between admission venous glucose level and 3-year risk (cumulative incidence) of type 2 diabetes among patients without known diabetes following an emergency admission to hospital. Similarly, as an additional outcome, we examined the association between admission glucose and mortality.

## Methods

### Ethics Statement

We obtained advice from the Research Ethics Committee (available from the author on request) that the research did not require ethical approval as this was a secondary analysis of routine anonymised data. Access to data for the purpose of performing this research was approved by NHS Information Services Division Privacy Advisory Committee (Ref 63/12) and the Caldicott Officer of each NHS Health Board in Scotland (via the Scotland-wide approval scheme).

### Patients

Using a unique identifier we performed a novel linkage of data on hospital admissions from the Scottish Morbidity Record (SMR01), laboratory results from SCI-Store, and diagnosis of diabetes from SCI-DC for three large Scottish Health Boards (Fife, Greater Glasgow and Clyde, and Lothian). Together these three Health Boards provide health care to approximately 2.4 million people, 46% of the Scottish population (General Registrar Office Scotland).

All patients aged 30 years or older who required emergency hospitalisation to internal medicine or surgical specialties, and were resident in the Health Board area in which the hospital was sited were included. We had originally planned only to examine the risk of type 2 diabetes for patients aged 40 and older as type 2 diabetes is rare in patients below this age. At the review stage, however, it was noted that our cohort presents an opportunity to examine associations among younger patients, who, in the absence of risk factors such as family history, are not normally offered testing for type 2 diabetes, and so we extended the analyses to include the younger age group [32],[33]. As this analysis was suggested during peer review, and was not part of our original protocol, results for younger patients aged 30 to 39 are reported separately. The introduction of standardised recording and coding of blood results was introduced across Scotland gradually. Therefore, the start of the inclusion period varied from December 2004 to March 2008 according to the admitting hospital. Data were extracted from SCI-DC on 1st December 2011, and therefore to ensure a minimum of 3-years follow-up for all patients we included patients admitted up to the 31st of November 2008. Where a patient had more than one admission during the study period the first was selected.

Prevalent diabetes was defined in patients with type 1 or type 2 diabetes diagnosed prior to the index admission or within 30 days of discharge, as recorded in SCI-DC. Patients with prevalent diabetes were excluded, as were those who died prior to discharge from hospital.

Having already completed our data analysis we unexpectedly were able to obtain data for an additional Scottish Health Board, NHS Tayside, which has a population of approximately 300,000. Hence, we used these additional data as a validation cohort.

### Outcomes

Incident type 2 diabetes was defined as having type 2 diabetes recorded on SCI-DC between 31 days and 3 years after the date of discharge from hospital. This definition was chosen to ensure that those patients who were admitted to hospital with a new diagnosis of diabetes, but in whom the diagnosis was not confirmed until a subsequent visit to primary care or an out-patient clinic, were not designated as incident cases. We chose 3 years as we felt that this was a reasonable compromise between selecting a timescale that was relevant for clinical decision making, while also allowing detection of most incipient cases of diabetes.

SCI-DC is a real-time single electronic record that is used to deliver and document all diabetes-related health care in Scotland. Clinical and administrative data are recorded for all diabetes care occurring in primary and secondary care (including physician, podiatry, dietetics, laboratory, and retinopathy services) for all adults and children with diabetes. SCI-DC is implemented across all NHS regional (Health Boards) within Scotland, and includes 99% of people resident in Scotland diagnosed with diabetes [31].

We also obtained outcome data on mortality from National Records Scotland (formerly General Registrar Office Scotland), which includes deaths occurring outside Scotland but within the UK. Deaths were defined as vascular if the underlying cause of death from the death certificate was coded, according to the tenth revision of International Classification of Diseases (ICD-10), as ischaemic heart disease (I20–I25), stroke (I63, I64, and I67), or other vascular diseases (I70–I79). Deaths were otherwise defined as non-vascular.

### Exposure

The first glucose measurement within 2 days of admission was defined as the admission glucose. A very small number of patients without prevalent diabetes (*n* = 122) had an admission glucose of >20 mmol/l and were excluded from all further analyses as these patients were assumed to have prevalent diabetes not recorded in the SCI-DC database owing to error.

### Covariates

Data on age, sex, and quintiles of the 2009 version of the Scottish Index of Multiple Deprivation (SIMD, an area-based measure of socio-economic deprivation) was obtained from the SMR01 database of hospitalisations. Specific diseases were defined if the primary cause of admission was coded (using ICD-10) as MI (I20–I25), stroke (G45 and I60–I67), COPD (J40–J44), or fracture (S02, S12, S22, S32, S42, S52, S62, S72, S82, and S92). The Charlson Index was calculated using the primary diagnosis recorded on SMR01 for all admissions during the five years prior to the index admission. Raised white cell count defined as ≥11×10^9^/l was included as a marker of acute stress.

### Statistical Analyses

Summary statistics for baseline characteristics were compared by category of admission glucose. As described in our pre-stated analysis plan (Protocol S1), we present the risk of diabetes across the range of glucose levels, which is a continuous measurement. Nevertheless, for illustration, cut-points for admission glucose were also chosen on the basis of the WHO criteria for defining categories of glucose tolerance, with an additional cut-off to indicate very high glucose: <6.1, 6.1–6.9, 7.0–11.0, 11.1–14.9, and ≥15 mmol/l for fasting/non-fasting samples [34].

The odds of type 2 diabetes within 3 years from discharge was modelled using logistic regression. Glucose was log-transformed for all regression analyses as a linearizing transformation. A priori we expected non-linear associations and so included polynomial terms in all models. We had originally planned to use non-parametric smoothers to allow for non-linearity, but used polynomials instead as these allow easier calculation of predicted values. The predicted 3-year risk of type 2 diabetes by level of admission glucose and covariates was obtained from these models. Discrimination was calculated as the area under the receiver operator characteristic curve (C-Statistic) with confidence intervals obtained via bootstrapping for both the development and validation cohorts using coefficients obtained from the model derived from the development cohort.

Interaction terms were used to examine whether associations differed for pre-specified sub-groups (admissions to medical versus surgical admissions, admission to intensive care units [ICUs], and admissions with principal diagnoses of MI, stroke, COPD, and fracture) and raised white cell count (as a marker of acute stress).

In sensitivity analyses we repeated the analyses for the sub-group of patients in whom there was 5 years of follow-up. In additional exploratory analyses we plotted the cumulative incidence of diabetes and death according to admission glucose (via estimating the sub-distribution cumulative incidence function for each stratum) using the entire follow-up time available for each patient, which ranged from 3 to 6.9 years. For this follow-up period, we examined associations between mortality and admission glucose using Cox proportional hazards models. We also modelled the cause-specific hazard ratio (csHR) for incident type 2 diabetes using Cox proportional hazard models, treating all-cause death as a censoring event, and modelled the sub-distributional hazard ratios (sdHRs) of type 2 diabetes using Fine and Gray models [35] (the most widely used method for modelling competing risks for time to event data) treating death as a competing risk.

All analyses were performed in R version 3.0.1 (R Project for Statistical Computing). Cox regression models and Fine and Gray models were fit using the survival and cmprsk packages, respectively [36],[37].

### Systematic Review

We completed a brief systematic review addressing the research question “What is the risk of type 2 diabetes according to admission glucose in acutely unwell hospitalised patients?” The search was performed on the 14th of October 2013. Table S1 reports the search terms. We included studies published in English on or after January 2000 for adults who had a non-elective hospital admission in whom diabetes status was assessed following discharge from hospital, and where the risk of type 2 diabetes was reported according to glucose level within 48 hours of admission. Studies that only reported the admission glycaemic status defined on the basis of an oral glucose tolerance test (OGTT) were excluded. We did not exclude studies that only reported fasting and not random admission glucose. Details of each study were tabulated. No meta-analysis was performed.

## Results

### Baseline Characteristics

**Figure 1:**
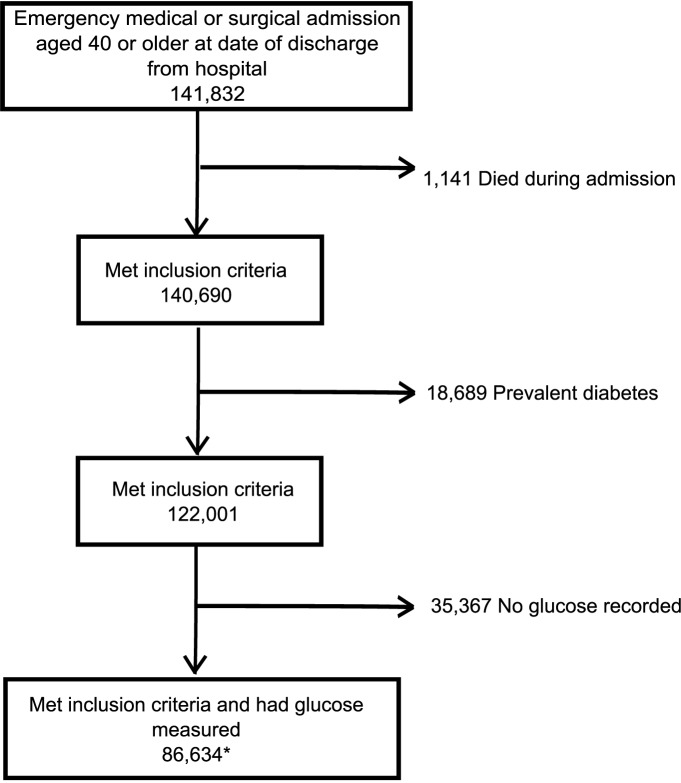
CONSORT style flowchart of patients included in analysis. * This group of 86,634 patients includes the *n* = 122 whose glucose was >20 mmol/l and so were assumed to have prevalent diabetes.

A total of 141,831 patients aged 40 and older met the inclusion criteria. Of these, 1,141 (0.8%) died during admission and 18,689 (13.2%) had prevalent diabetes and so were excluded. A total of 86,634 (71.0%) of the remaining 122,001 had a glucose measure within 2 days of admission (Figure 1).

Patients admitted to medical specialties were more likely to have glucose measured than those admitted to surgical specialties (62,508 [80.8%] versus 28,356 [56.9%]) as were those with MI, stroke, and COPD (89.9%, 89.6%, and 86.0%, respectively), and those older than 80. Glucose measurement was not, however, related to sex or socio-economic deprivation (Table 1). The 3-year risk of diabetes was similar in patients with and without glucose measured on admission (2.3% [1,965/84,669] versus 2.0% [725/34,642]).

**Table 1:** Glucose measurement on admission by patient characteristics.

Variable	Value	Number (%)^a^
Total		86,634 (71.0)
Age (years)	40 to 49	15,786 (70.2)
	50 to 59	15,324 (70.4)
	60 to 69	16,107 (68.5)
	70 to 79	18,989 (69.6)
	>80	20,428 (75.8)
Sex	Women	45,972 (69.6)
	Men	40,662 (72.7)
SIMD	Quintile 1 (most deprived)	25,012 (71.5)
	Quintile 2	18,812 (71.9)
	Quintile 3	13,221 (71.2)
	Quintile 4	12,106 (70.1)
	Quintile 5 (least deprived)	17,483 (69.9)
Specialty	Medicine	62,508 (80.8)
	Surgery	28,356 (56.9)
	ICU	1,853 (81.8)
Diagnostic sub-groups	MI	6,215 (89.9)
	Stroke	3,753 (89.6)
	COPD	3,016 (86)
	Fracture	5,061 (63.9)
Hospital		68.5 (64.5–74.1) [22.6]

a All figures are counts (%) except for hospital, for which the proportion with glucose measures across hospitals was summarised using the median (interquartile range) and [range].

Patients with glucose measures were on average 66.3 years old and 40,596 (46.9%) were male. As expected for a hospitalised population, people resident in more socio-economically deprived areas were overrepresented (Table 2).

**Table 2:** Baseline characteristics by admission glucose.

Patient Characteristics/Glucose (mmol/l)	<6.1	6.1–6.9	7.0–11.0	11.1–14.9	≥15	Glucose Measures	Glucose Not Measured	Validation Cohort
N	46,499	18,185	20,030	1,480	318	86,512	35,367 (100)	22,655
Age, years	64.3 (15.3)	67.8 (14.8)	69.5 (14.4)	69.4 (14.0)	68.7 (13.8)	66.3 (15.1)	65 (14)	67 (15)
Male	21,897 (47.1)	8,531 (46.9)	9,273 (46.3)	743 (50.2)	152 (47.8)	40,596 (46.9)	15,262 (43.2)	10,690 (47.2)
SIMD quintile								
Q1 (most deprived)	13,738 (29.5)	5,107 (28.1)	5,560 (27.8)	468 (31.6)	89 (28.0)	24,962 (28.9)	9,961 (28.2)	5,695 (25.1)
Q2	10,114 (21.8)	3,956 (21.8)	4,317 (21.6)	326 (22.0)	73 (23.0)	18,786 (21.7)	7,368 (20.8)	4,481 (19.8)
Q3	6,904 (14.8)	2,844 (15.6)	3,168 (15.8)	238 (16.1)	53 (16.7)	13,207 (15.3)	5,351 (15.1)	4,073 (18.0)
Q4	6,477 (13.9)	2,600 (14.3)	2,758 (13.8)	207 (14.0)	45 (14.2)	12,087 (14)	5,154 (14.6)	5,148 (22.7)
Q5 (least deprived)	9,266 (19.9)	3,678 (20.2)	4,227 (21.1)	241 (16.3)	58 (18.2)	17,470 (20.2)	7,533 (21.3)	3,258 (14.4)
Elevated WCC	10,817 (23.3)	6,481 (35.6)	9,141 (45.6)	814 (55.0)	195 (61.3)	27,448 (31.7)	8,377 (23.7)	7,003 (31.5)
Charlson index								
0	30,482 (65.6)	11,290 (62.1)	12,071 (60.3)	787 (53.2)	140 (44.0)	54,770 (63.3)	23,039 (65.1)	29,655 (61.1)
1–4	3,665 (7.9)	1,519 (8.4)	1,666 (8.3)	164 (11.1)	44 (13.8)	7,058 (8.2)	2,167 (6.1)	4,046 (8.3)
5–6	1,985 (4.3)	976 (5.4)	1,117 (5.6)	86 (5.8)	24 (7.5)	4,188 (4.8)	1,084 (3.1)	2,555 (5.3)
>6	10,367 (22.3)	4,400 (24.2)	5,176 (25.8)	443 (29.9)	110 (34.6)	20,496 (23.7)	9,077 (25.7)	12,267 (25.3)
Medical specialty	32,863 (70.7)	12,978 (71.4)	13,990 (69.8)	1,084 (73.2)	238 (74.8)	61,153 (70.7)	14,901 (42.1)	35,211 (72.6)
ICU	598 (1.3)	320 (1.8)	702 (3.5)	148 (10.0)	60 (18.9)	1,828 (2.1)	411 (1.2)	1,176 (2.4)
MI	2,984 (6.4)	1,304 (7.2)	1,687 (8.4)	177 (12.0)	52 (16.4)	6,204 (7.17)	698 (2.0)	4,002 (8.2)
Stroke	1,929 (4.1)	819 (4.5)	890 (4.4)	74 (5.0)	11 (3.5)	3,723 (4.3)	437 (1.2)	2,315 (4.8)
COPD	1,376 (3.0)	713 (3.9)	814 (4.1)	86 (5.8)	14 (4.4)	3,003 (3.47)	492 (1.4)	1,479 (3.0)
Fracture	2,162 (4.6)	1,274 (7.0)	1,472 (7.3)	61 (4.1)	7 (2.2)	4,976 (5.75)	2,853 (8.1)	656 (2.9)

All data are *n* (%) except age, which is reported as mean (standard deviation).

Elevated WCC, white cell count ≥11×10^9^/l.

### Distribution of Glucose

Admission glucose was <6.1 mmol/l for 53.7% (46,499/86,512) of patients, and was between 6.1 and 6.9 mmol/l for 21.0% (18,185/86,512), ≥7 mmol/l for 25.3% (21,828/86,512), and ≥11.1 mmol/l for 2.1% (1,798/86,512) of patients. For patients with MI, COPD, and fracture the percentage with glucose ≥7 mmol/l was approximately 5% higher than for patients overall (30.8% [1,916/6,204], 30.4% [917/3012], and 31.1% [1,572/5,057], respectively).

Patients with higher glucose levels were on average older and were more likely to have been admitted to an ICU than those with lower levels. Co-morbidity (assessed via Charlson Index) and a raised white cell count was also more common in patients with elevated glucose (Table 2).

### 3-Year Risk of Type 2 Diabetes

The 3-year risk of type 2 diabetes was 2.3% (1,952 of 86,512). The 3-year risk, by category of glucose, age, and sex is presented in Table 3.

**Table 3:** 3-year risk of type 2 diabetes stratified by admission glucose, age, and sex.

Sex	Age (y)/Glucose (mmol/l)	<6.1	6.1–6.9	7.0–11.0	11.1–14.9	≥15
Men	30–39	10 (0.3)	10 (1.0)	22 (2.7)	4 (6.5)	4 (30.8)
	40–69	198 (1.4)	154 (3.1)	321 (6.2)	77 (18.5)	24 (27.6)
	70–79	52 (1.2)	39 (1.9)	109 (4.7)	35 (18.3)	5 (12.5)
	80 and older	17 (0.5)	9 (0.6)	33 (1.8)	9 (6.7)	1 (4)
Women	30–39	12 (0.3)	3 (0.4)	13 (2.1)	3 (8.3)	3 (33.3)
	40–69	133 (1)	86 (2.1)	206 (5.1)	52 (18.9)	13 (18.3)
	70–79	54 (1.1)	47 (2)	113 (4.1)	18 (9.8)	4 (9.5)
	80 and older	21 (0.3)	24 (0.8)	77 (2.0)	19 (6.8)	2 (3.8)

All data are n (%).

**Figure 2:**
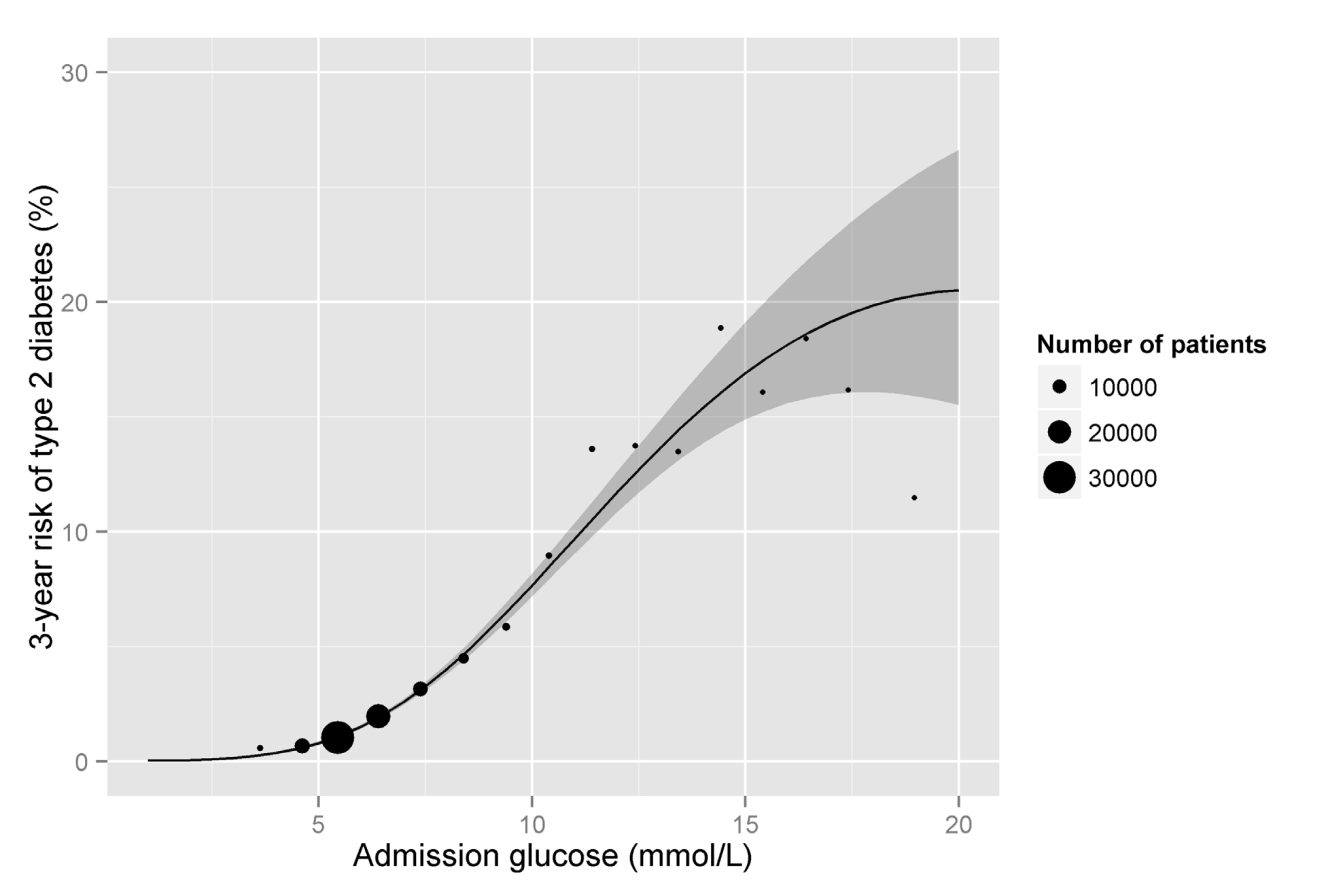
3-year risk of type 2 diabetes by admission glucose. The solid line represents the estimate and the ribbon represents the 95% CI obtained from a logistic regression model of incident type 2 diabetes on glucose, glucose-squared, and glucose-cubed. Points represent the risk of diabetes for patients categorised according to admission glucose, with the x-axis indicating the mean glucose level and the point size indicating the number of patients for each category.

In logistic regression models, the 3-year risk of type 2 diabetes was strongly associated with admission glucose (Figure 2). From a very low risk of diabetes with glucose levels <5 mmol/l the risk increased linearly to 15 mmol/l before reaching a plateau. The 3-year risks of diabetes at the WHO cut-points for diagnosing diabetes on fasting (7 mmol/l) and random (11.1 mmol/l) samples were 2.6% (95% CI 2.5–2.7) and 9.9% (95% CI 9.2–10.6), respectively, with a risk of 5.7% at 9 mmol/l.

**Figure 3:**
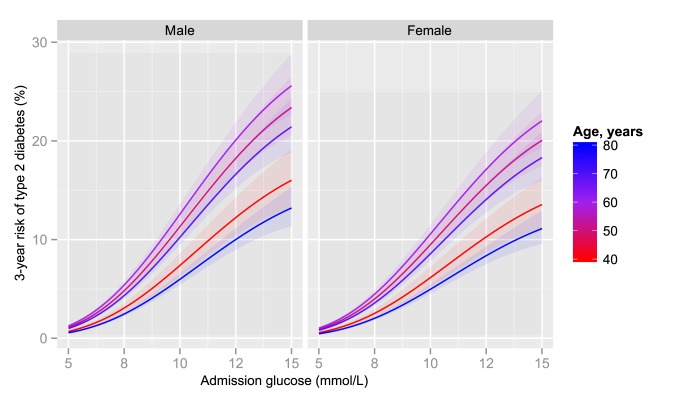
3-year risk of type 2 diabetes by admission glucose, age, and sex. The solid lines represent the estimates with ribbons indicating 95% CIs obtained from a logistic regression model of incident type 2 diabetes on glucose, glucose-squared, and glucose-cubed, adjusting for age and sex.

At every level of glucose, the 3-year risk of diabetes was higher in men than women. The association with age was non-linear (Figure 3), with the highest risk of diabetes among patients aged 50 to 69 with lower risks for both younger and older patients. The risk was particularly low for those over 80 years of age. Socio-economic deprivation was strongly associated with an increased risk of diabetes (odds ratio for least deprived versus most deprived quintile 0.74; 95% CI 0.64–0.85) (Table 4). There was no statistically significant evidence of interaction with glucose on a multiplicative scale for age, sex, or SIMD: the odds ratio (OR) for interaction was 0.94 (95% CI 0.86–1.02, *p* = 0.13) per ten years of age, 1.11 (95% CI 0.90–1.36, *p* = 0.33) for sex, and 1.00 (95% CI 0.94–1.08, *p* = 0.89) per 1 quintile increment in SIMD.

**Table 4:** 3-year risk of type 2 diabetes by glucose, age, sex, and Scottish Index of Multiple Deprivation (SIMD) score: coefficients and odds ratios from logistic regression models.

Variable in Model	Model 1		Model 2	
	Coefficient	OR (95% CI)	Coefficient	OR (95% CI)
Intercept	−13.052		−13.052	
Age, per ten years, squared	2.052	7.78 (5.50–11.00)	2.05	7.77 (5.49–10.98)
Age, per ten years, squared	−0.176	0.84 (0.82–0.86)	−0.175	0.84 (0.82–0.86)
Women	−0.196	0.82 (0.75–0.90)	−0.2	0.82 (0.75–0.90)
Glucose	−2.375	0.09 (0.07–0.13)	−2.356	0.09 (0.07–0.13)
Glucose squared	3.723	41.40 (24.65–69.50)	3.719	41.22 (24.53–69.27)
Glucose cubed	−0.749	0.47 (0.41–0.54)	−0.75	0.47 (0.41–0.54)
SIMD Quintile 1 (most deprived)	—	—	0.298	1.35 (1.17–1.55)
SIMD Quintile 2	—	—	0.301	1.35 (1.17–1.57)
SIMD Quintile 3	—	—	0.137	1.15 (0.97–1.35)
SIMD Quintile 4	—	—	0.079	1.08 (0.91–1.29)
SIMD Quintile 5 (least deprived)	—	—	0	1

There were no statistically significant interactions (on the scale of the linear predictor) for any of these variables. The natural logarithm of glucose in mmol/l was used for all models. Glucose provided in mg/dl should be converted to mmol/l by dividing by 18. The 3-year risk of type 2 diabetes is the inverse logit of the linear predictor (sum of the relevant coefficients). For example, for a male patient aged 60 with a glucose of 5 mmol/l the risk of type 2 diabetes is calculated as follows:
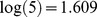


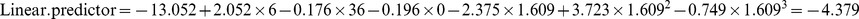


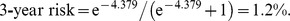

Fitted values with standard errors and the model variance/covariance matrix are shown in Tables S8 and S9. We have also provided an online tool to carry out this calculation, which is available at www.cphs.mvm.ed.ac.uk/diabetes-risk/.

In models adjusting for age, sex, and admission glucose, neither speciality (medical OR 1.09; 95% CI 0.98–1.21, *p* = 0.10), elevated white cell count (OR 0.93; 95% CI 0.80–1.08, *p* = 0.31) nor co-morbidity (Charlson Index greater than zero, OR 1.04; 95% CI (0.94–1.14), *p* = 0.48) were associated with 3-year risk of diabetes. Nor was there statistically significant evidence of interaction on a multiplicative scale between these variables and admission glucose: the OR for interaction was 0.85 (95% CI 0.67–1.06, *p* = 0.16) for medical specialty, 0.83 (95% CI 0.58–1.13, *p* = 0.27) for elevated white cell count, and 0.89 (95% CI 0.73–1.10, *p* = 0.29) for Charlson Index greater than zero.

### Sub-group Analyses

**Figure 4:**
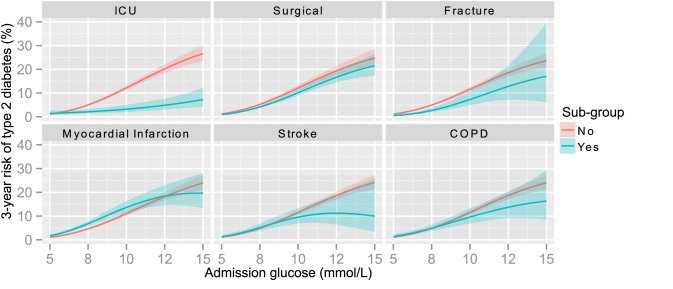
3-year risk of type 2 diabetes by glucose for patients in sub-groups. Predicted 3-year risks of type 2 diabetes by glucose level obtained from logistic regression models. All models adjust for age, sex, and a main term and interaction term with glucose for the relevant grouping variable (e.g., admission to ICU). Lines represent estimates and ribbons indicate 95% CIs with blue used to indicate membership of the relevant sub-group and red used to describe the remainder of the population.

Associations for each sub-group, examined via adding interaction terms with glucose, are shown in Figure 4. The overall risk of diabetes for patients admitted to ICU was lower than the general population, and the association between hyperglycaemia and risk of type 2 diabetes was weaker in both absolute and relative terms (Table 5). The risks were similar for surgical and medical specialties, however, and for each of the groups defined by pre-specified diagnoses (Figure 4). Similar results were obtained in stratified analyses (Figure S1).

**Table 5:** Predicted 3-year risk of type 2 diabetes in patients admitted to ICU compared to patients not admitted to ICU.

Glucose mmol/l	Not Admitted ICU		Admitted ICU	
	Risk (95% CI)	Risk Difference (Risk Ratio)	Risk (95% CI)	Risk Difference (Risk Ratio)
5	1.09 (0.95–1.24)	reference	1.40 (0.72–2.71)	reference
10	11.75 (10.62–12.97)	10.66 (10.77)	3.17 (1.98–5.02)	1.77 (2.26)
15	25.69 (22.34–29.36)	24.6 (23.57)	7.13 (4.11–12.10)	5.73 (5.09)

Estimates and 95% CIs were obtained from logistic regression models with ICU as an interaction term. The reference category for each risk ratio and risk difference was the risk for patients with admission glucose equal to 5 mmol/l.

### Prediction

Discrimination for admission glucose in predicting 3-year risk of diabetes in the development cohort was good. For age, sex, and glucose the area under the receiver operator characteristic curve (C-statistic) was 0.75 (95% CI 0.74–0.76), which was an improvement over the C-statistic for age and sex alone which was 0.62 (95% CI 0.61–0.64).

**Figure 5:**
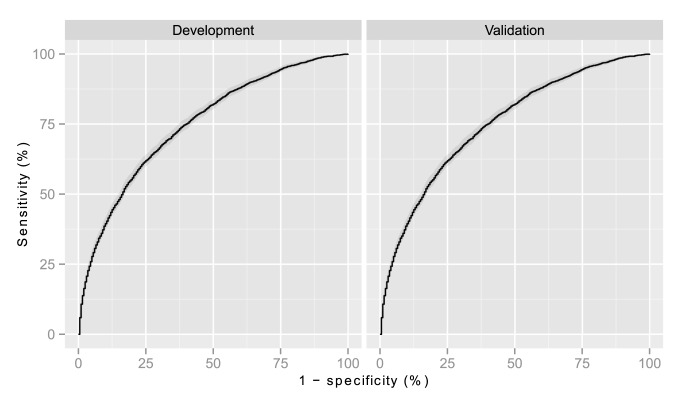
Receiver operator characteristic curve for development and validation cohorts. The ribbon represents the 95% CIs.

The independent validation cohort comprised the 22,655 patients with an emergency medical or surgical hospital admission to Ninewells hospital in NHS Tayside from 2005 to 2008. Laboratory data were provided for this cohort by the Health Informatics Centre, University of Dundee. The patient characteristics were similar to that of the development cohort (Table 2). In this cohort the C-statistic for glucose, age, and sex was 0.75 (95% CI 0.73–0.76), so there was no evidence of over-fitting (Figure 5).

### Mortality

Follow-up time for the entire cohort of 86,512 patients aged 40 and older comprised 297,122 person-years (median 3.6, interquartile range 2.7–4.7, and maximum 6.9 person-years) during which there were 2,724 cases of incident diabetes (9.2 per 1,000 person-years) and 25,193 deaths (85.8 per 1,000 person-years), of which 2,406 (8.1 per 1,000 person-years) were due to vascular disease.

**Figure 6:**
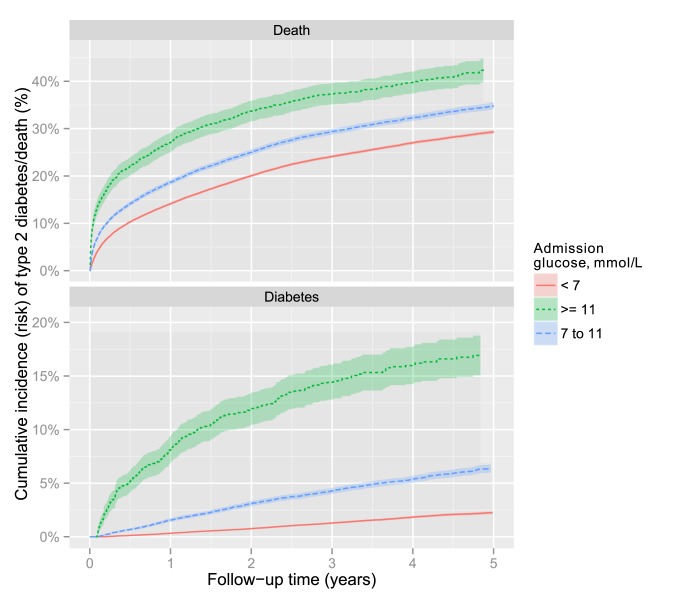
Cumulative incidence of mortality and type 2 diabetes by admission glucose. Non-parametric estimates of cumulative incidence of mortality and incident type 2 diabetes according to admission glucose. The ribbon represents the 95% CIs.

Admission glucose was associated with increased mortality rates as well as with increased risk of type 2 diabetes. The mortality rate was 7.48 per 100 person-years (12,315/164,548) for patients with a glucose level <6.1 mmol/l compared to 13.9 (603/4,337) for patients with a glucose level of 11.1–14.9 mmol/l (Table 6). However, the association was considerably stronger for diabetes (Figure 6). Patients with glucose levels of 11.1–15 mmol/l and >15 mmol/l had higher mortality than patients with a glucose of <6.1 mmol/l (hazard ratio [HR] 1.54; 95% CI 1.42–1.68 and 2.50; 95% CI 2.14–2.95, respectively) in models adjusting for age and sex. Stronger associations were found for deaths due to vascular disease than for other causes of death. For example, compared to patients with glucose <6.1 mmol/l those with glucose in the 11.1–14.9 mmol/l range had higher vascular (HR 2.61; 95% CI 2.12–3.20) than non-vascular mortality rates (HR 1.34; 95% CI 1.22–1.46). Table 6 presents mortality results for glucose modelled both as a categorical and a continuous variable, for both unadjusted and adjusted models.

**Table 6:** Mortality according to admission glucose.

Patient Characteristics/Glucose (mmol/l)	<6.1	6.1–6.9	7.0–11.0	11.1–14.9	≥15
*N*	46,499	18,185	20,030	1,480	318
Person-years	164,548	61,761	65,651	4,337	826
All deaths, *n*	12,315	5,459	6,662	603	154
Mortality rate, per 100 person-years	7.48	8.84	10.15	13.90	18.64
HR, unadjusted	1	0.99 (0.96–1.02)	1.06 (1.03–1.09)	1.54 (1.42–1.68)	2.50 (2.14–2.93)
HR, model 1	1	1.17 (1.13–1.21)	1.36 (1.32–1.40)	1.96 (1.81–2.13)	2.74 (2.33–3.21)
HR, model 2	1	0.99 (0.96–1.02)	1.07 (1.04–1.10)	1.45 (1.34–1.58)	2.29 (1.95–2.68)
HR, model 2, continuous^a^					
Vascular deaths, *n*	1,060	506	708	100	32
HR, unadjusted	1	1.03 (0.92–1.14)	1.25 (1.13–1.37)	2.78 (2.26–3.41)	5.65 (3.97–8.03)
HR, model 1	1	1.26 (1.13–1.40)	1.66 (1.51–1.83)	3.68 (3.00–4.52)	6.31 (4.44–8.97)
HR, model 2	1	1.04 (0.93–1.15)	1.27 (1.15–1.40)	2.61 (2.12–3.20)	4.89 (3.44–6.95)
HR, model 2, continuous^b^					
Non-vascular deaths, *n*	11,255	4,953	5,954	503	122
HR, unadjusted	1	0.98 (0.95–1.02)	1.04 (1.01–1.08)	1.42 (1.30–1.55)	2.18 (1.83–2.61)
HR, model 1	1	1.16 (1.12–1.20)	1.33 (1.29–1.37)	1.79 (1.64–1.96)	2.38 (1.99–2.85)
HR, model 2	1	0.98 (0.95–1.02)	1.05 (1.02–1.08)	1.34 (1.22–1.46)	2.01 (1.68–2.40)
HR, model 2, continuous^c^					

Hazard ratio (95% CI). Model 1 adjusts for age, age-squared, and sex. Model 2 additionally adjusts for SIMD quintile, comorbidity (Charlson Index), COPD, and medical speciality.

Results shown for glucose categorised and modelled as a polynomial.

a Glucose 1.16 (1.07–1.25). Glucose-squared 0.42 (0.36–0.48) and glucose cubed 1.35 (1.29–1.41).

b Glucose 1.24 (0.93–1.66). Glucose-squared 0.41 (0.27–0.63) and glucose cubed 1.40 (1.25–1.58).

c Glucose 1.15 (1.05–1.24). Glucose-squared 0.43 (0.37–0.50) and glucose cubed 1.33 (1.27–1.39).

### Competing Risks

**Figure 7:**
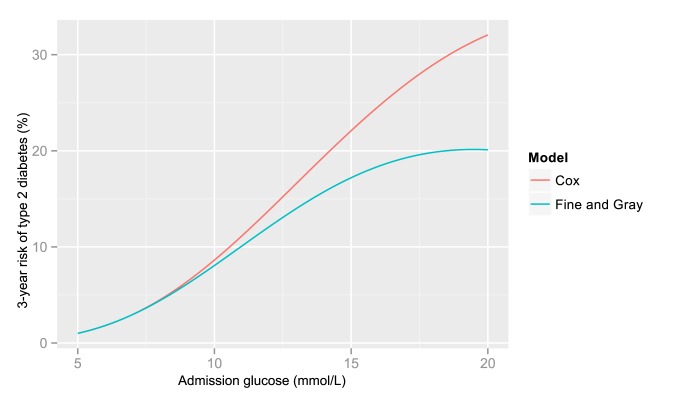
3-year risk of type 2 diabetes and competing risks. Estimates of 3-year risk of type 2 diabetes in women aged 60 obtained by multiplying the risk in women aged 60 with a glucose of 5 mmol/L (1.0%, obtained from the logistic regression model in Table 4) by the csHR and the sdHR, respectively.

In exploratory time to event analyses, death did not appear to be an important competing risk in terms of the association between admission glucose and diabetes. Over 297,122 person-years of follow-up (median 3.6, interquartile range 2.7–4.7, and maximum 6.9 person-years) there were 25,193 deaths and 2,724 cases of incident diabetes. The csHR (where death is treated as a censoring event) and the sdHR (where death is treated as a competing event) were similar: for example the csHR and sdHR for patients with a glucose of 11.1–14.9 mmol/l (compared to a glucose of <6.1 mmol/l) were 14.1 and 12.0, respectively (Table 7). Figure 7 presents the predicted risks based on the csHRs and sdHRs across the range of glucose levels. For glucose levels <15 mmol/l, the risk of diabetes was very similar for both models. For glucose levels ≥15 mmol/l the risks estimated from the csHRs were higher than those from the sdHRs. Nevertheless, both models resulted in predicted 3-year risks of diabetes above 18% for glucose levels ≥15 mmol/l.

**Table 7:** Diabetes according to admission glucose from competing risks analysis.

Patient Characteristics/Glucose (mmol/l)	<6.1	6.1–6.9	7.0–11.0	11.1–14.9	≥15
*N*	46,499	18,185	20,030	1,480	318
Person-years	164,548	61,761	65,651	4,337	826
Diabetes events	768	514	1,150	241	51
csHR	1	1.80; 95% CI (1.61–2.01)	3.89; 95% CI (3.55–4.26)	14.08; 95% CI (12.18–16.28)	16.68; 95% CI (12.56–22.15)
sdHR	1	1.82; 95% CI (1.63–2.04)	3.88; 95% CI (3.54–4.26)	12.02; 95% CI (10.36–13.96)	11.55; 95% CI (8.56–15.60)
csHR, continuous^a^					
sdHR, continuous^b^					

csHR (95% CI) derived from Cox regression model. sdHR (95% CI) derived from Fine and Gray regression model. All models adjust for age, age-squared, and sex. Results shown for glucose categorised and modelled as a polynomial.

a Glucose 0.15 (0.11–0.19): Glucose-squared 18.33 (12.05–27.87): and glucose cubed 0.58 (0.52–0.65).

b Glucose 0.15 (0.11–0.20): Glucose-squared 22.74 (14.58–35.45): and glucose cubed 0.53 (0.47–0.60).

Very similar results were found in the sub-group of patients who had been admitted to ICU (Table S2).

Death did not appear to be a competing risk in terms of the association between age and diabetes either. Indeed, the sdHR for age and age-squared was higher than the csHR (Table 8).

**Table 8:** Diabetes according to age, sex, and admission glucose from time to event analyses.

Variable in Model	csHR	sdHR
Age, per ten years	6.92 (5.15–9.29)	9.17 (6.86–12.26)
Age, per ten years, squared	0.85 (0.83–0.87)	0.83 (0.81–0.85)
Male	0.79 (0.73–0.86)	0.83 (0.77–0.90)
Glucose, mmol/l	0.15 (0.11–0.19)	0.15 (0.11–0.20)
Glucose, mmol/l, squared	18.33 (12.05–27.87)	22.74 (14.58–35.45)
Glucose, mmol/l, cubed	0.58 (0.52–0.65)	0.53 (0.47–0.60)

csHR (95% CI) derived from Cox regression model. sdHR (95% CI) derived from Fine and Gray regression model. Estimates are presented for all variables included in the models.

### Younger Patients

In a post hoc analysis requested during the peer review process, we examined associations for patients aged 30 to 39 years old. A total of 18,643 patients in this age-group had an emergency admission to hospital during the study period and met the inclusion criteria. Of these, 13 (0.07%) died during admission and 658 (3.5%) had prevalent diabetes and so were excluded. A total of 11,889 (66.2%) of the remaining 17,972 had a glucose measure within 2 days of admission. A total of 14 patients had a glucose of >20 and were excluded from all further analyses.

Compared to patients aged ≥40 years, patients aged 30 to 39 years were more likely to have a high white cell count (35.0% versus 31.7%), to be admitted to surgical specialties, and to reside in an area with high deprivation than were older patients (Table S3). COPD, MI, stroke, and comorbidity were very uncommon in this group (all <1%).

Admission glucose was above the conventional cut-points around half as often in younger patients compared to older patients. Only 1,588 (13.1%) of younger patients had glucose ≥7 mmol/l and 120 (1%) of younger patients had glucose ≥11.1 mmol/l (Table S4). The 3-year risk of type 2 diabetes was 0.7% (84/11,875) overall.

In logistic regression models, similar associations were observed for admission glucose and 3-year risk of type 2 diabetes as were seen among older patients (Table 9). Consistent with the lower overall risk, the 3-year risks of diabetes at the WHO cut-points for diagnosing diabetes on fasting (7 mmol/l) and random (11.1 mmol/l) samples were low: 1.0% (95% CI 0.8–1.3) and 7.8% (95% CI 5.7–10.7), respectively, with a risk of 3.3% at 9 mmol/l.

**Table 9:** 3-year risk of type 2 diabetes by glucose, age, sex, and Scottish Index of Multiple Deprivation (SIMD) score: coefficients and odds ratios from logistic regression models for patients aged 30–39.

Variable in Model	Model 1		Model 2	
	β	OR (95% CI)	β	OR (95% CI)
Intercept	−11.843		−11.338	
Age, per ten year	0.085	1.09 (1.00–1.18)	0.087	1.09 (1.01–1.18)
Women	−0.145	0.86 (0.55–1.35)	−0.118	0.89 (0.57–1.39)
Glucose	−3.078	0.05 (0.01–0.17)	−2.839	0.06 (0.02–0.22)
Glucose squared	4.164	64.30 (7.83–528)	3.727	41.55 (4.88–354.11)
Glucose cubed	−0.738	0.48 (0.27–0.83)	−0.616	0.54 (0.31–0.95)
SIMD Quintile 1 (most deprived)	—	—	−0.095	0.91 (0.53–1.56)
SIMD Quintile 2	—	—	−0.525	0.59 (0.30–1.18)
SIMD Quintile 3	—	—	−1.134	0.32 (0.12–0.83)
SIMD Quintile 4	—	—	−1.029	0.36 (0.15–0.83)
SIMD Quintile 5 (least deprived)	—	—	0.087	1.09 (1.01–1.18)

The natural logarithm of glucose in mmol/l was used for all models.

### Sensitivity Analyses

**Figure 8:**
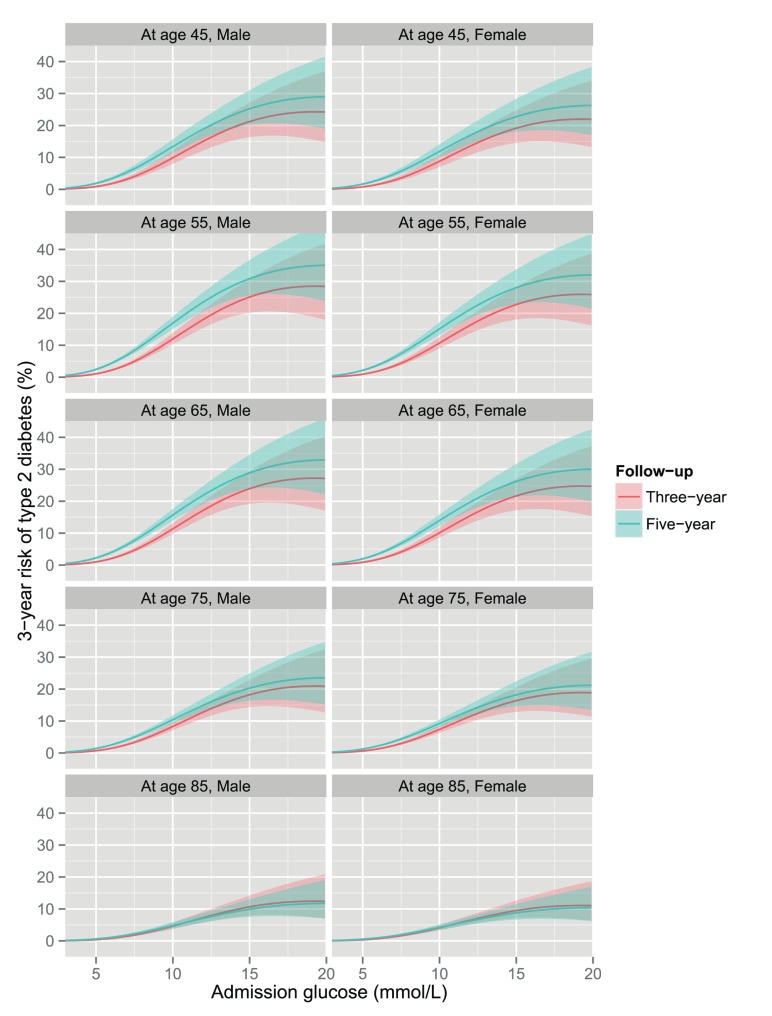
3-year risk and 5-year risk of type 2 diabetes by admission glucose among patients with 5 or more years of follow-up. The solid lines represent the predicted risk with ribbons indicating the 95% CIs obtained from a logistic regression model of each outcome on glucose, glucose-squared, and glucose-cubed, adjusting for age and sex.

Of the 27,190 patients with five or more years of follow-up, 932 (3.4%) developed type 2 diabetes within 5 years. Across age and sex strata the 5-year risk of type 2 diabetes according to admission glucose level was similar to the 3-year risk (Figure 8).

### Systematic Review

From the 2,555 titles identified 2,514 irrelevant or duplicate articles were excluded. On full-text review, 13 of the 41 remaining articles met the selection criteria [14]–[20],[23],[24],[27],[38]–[40]. All but two studies were single centre and the majority recruited only patients with specific diseases, most commonly acute coronary disease or stroke. Inclusion/exclusion criteria were heterogeneous in terms of age and other risk factors, as were definitions of stress hyperglycaemia and subsequent diabetes. Only five studies had more than 12 weeks follow-up (range 2.5–5 years) and of these, with the exception of one study that followed up more than 95% of 2,215 people with community acquired pneumonia [20], the completeness of follow-up was low. The resultant estimates for subsequent risk of type 2 diabetes according to stress hyperglycaemia therefore varied from 7% to 36%, and results were not presented stratified by age and/or sex (Table S7).

## Discussion

Admission glucose is measured routinely on emergency hospitalisation, and is above the (symptomatic) cut-point for diagnosis of diabetes (7.0 mmol/l) in over one-quarter of patients. Clinicians are unclear as to the longer term significance of an elevated glucose. Our study is the first, to our knowledge, to report the long term risk of diabetes in this group of patients on a population level. We report that the 3-year risk of type 2 diabetes was <1% for patients with a glucose of ≤5 mmol/l and increased linearly to approximately 15% at 15 mmol/l, above which there was no further increase in risk. The 3-year risks at the WHO diabetes diagnostic cut-points for fasting (7 mmol/) and random (11.1 mmol/l) glucose [34] were 3% and 10%, respectively. One in four and one in 40 patients had a glucose level above these respective cut-points. For the first time, to our knowledge, these results will allow doctors to inform patients of their future risk of diabetes and will direct future guidelines on the appropriate follow-up of abnormal hospital glucose results.

Our findings confirm that single measures using the WHO cut-points for diagnosing diabetes, which are not based upon studies that measured glucose in the emergency setting [34], cannot be directly applied in acutely unwell patients with hyperglycaemia. Indeed, we found that 90% of patients with an admission glucose of 11.1 mmol/l were not diagnosed with diabetes within 3 years of discharge from hospital. This figure is likely to be lower in populations with a higher baseline risk of diabetes, but even for settings where the baseline risk of diabetes is 4-fold higher, we would still expect fewer than half of patients with an admission glucose of 11.1 mmol/l to be diagnosed with diabetes within 3 years. As such, for most countries and health care systems, clinicians treating patients in whom a high glucose is detected during an emergency hospital admission can be reassured that the most likely diagnosis is stress hyperglycaemia rather than type 2 diabetes.

Consistent with previous reports, we also found that high admission glucose was associated with increased mortality [1],[2],[4]–[8], and that the association was stronger for deaths related to vascular diseases compared to other causes of death. The association for mortality was, however, 2-fold to 5-fold weaker than the association for type 2 diabetes. This weaker association, in relative terms, may partly explain why death did not appear to be a “competing risk” for incident type 2 diabetes, that is increased deaths among patients with high blood glucose levels, compared to those with lower blood glucose levels, did not have an important impact on the relationship between admission glucose and incidence of type 2 diabetes.

Nevertheless, there were around six more deaths per 100 person-years among patients with glucose levels above 11.1 mmol/l, compared to patients with glucose <6.1 mmol/l, which is a large absolute difference in mortality rate. Moreover, the association with mortality was only slightly attenuated on adjusting for age, sex, socio-economic deprivation, comorbidity, COPD, and medical specialty. Consequently, patients with hyperglycaemia can be considered to be at considerably higher risk of both death and diabetes.

Previous follow-up studies have estimated the risk of type 2 diabetes among patients with hyperglycaemia [14]–[28]. In the best of these, a case-series of 2,215 patients in Alberta, Canada with community acquired pneumonia, the risk of type 2 diabetes was increased in 473 patients with moderate hyperglycaemia (7.8–11.0 mmol/l) and 104 patients with severe hyperglycaemia (11.1–20.0 mmol/l), but was not higher for mild hyperglycaemia (6.1–7.0 mmol/l) [20]. We found similar relative risks for moderate and severe hyperglycaemia. However, in a representative sample of more than 85,000 patients, in which approximately 2,000 developed incident diabetes over 3 years, we identified that the association between admission glucose and risk of type 2 diabetes was linear in the 5–15 mmol/l range. Consequently even mild hyperglycaemia is associated with increased risk of type 2 diabetes.

Our large sample size also allowed us to examine whether the association between risk of diabetes and glucose level differed according to age and sex. We found that men had a higher risk of diabetes at every level of glucose, while patients aged 50–69 years were at higher risk than both older and younger age groups.

We also examined diabetes risks according to admission glucose for pre-specified admission diagnoses and found similar associations to the main analyses for patients admitted with MI, COPD, stroke, and fracture. Current guidance for the management of patients with acute coronary syndrome suggest, on the basis of short-term follow-up studies [21],[22],[25],[26], that patients with hyperglycaemia without diabetes should be screened for diabetes at least annually [41]. This recommendation may be refined on the basis of the estimates of the risk of diabetes that we have provided.

We have provided a risk calculator to allow clinicians to estimate the age-sex specific 3-year risk of type 2 diabetes (Glucose on Unselected Admissions and Risk of Diabetes [GUARD]: Type 2 Diabetes Risk Calculator available at www.cphs.mvm.ed.ac.uk/diabetes-risk/). Since we obtained similar results for medical and surgical specialities, across a number of common clinical conditions, and for patients with and without comorbidity, this tool is likely fairly robust to moderate differences in the organisation of secondary care, and may therefore be used in settings that have similar age-sex specific risks of diabetes to those observed in Scotland [42].

Prediction scores have previously been developed to estimate the risk of developing type 2 diabetes in order to identify healthy people in whom glucose should be measured [29],[30]. Instead, our score was designed to aid clinicians faced with interpreting an abnormally high glucose level discovered following a routine panel of blood tests during admission to hospital, where it is uncertain whether stress hyperglycaemia or type 2 diabetes is the underlying cause. Nonetheless, the area under the curve we observed in our validation cohort (C-statistic 0.75) was comparable to that of non-biomarker based scores when externally validated in the Kora and Whitehall cohorts (C-statistics ranged from 0.62 to 0.68 [29] and 0.61 to 0.67 [30], respectively).

There is on-going controversy concerning screening for type 2 diabetes in the general adult population and for specific-disease groups, and professional and guideline bodies in different countries differ in their recommendations on which adults should be screened [32],[43],[44]. Notwithstanding these differences in care, since patients with an admission glucose level of 11.1 mmol/l have a 3-year risk of type 2 diabetes of 10% (5-fold higher than the risk in the general population), we propose that patients whose admission glucose is at or above this concentration should be offered follow-up testing. Since fewer than one in 40 patients had a glucose level ≥11.1 mmol/l, repeat testing should be achievable in most health care settings.

For lower glucose levels, our findings will allow clinicians to communicate to patients their risk of diabetes, and to provide simple lifestyle modification advice as appropriate. Ultimately, we hope that our findings will be incorporated into modelling studies comparing the cost-effectiveness of different strategies for identifying diabetes, such as population-based screening and opportunistic testing. One reason why opportunistic testing for type 2 diabetes in the hospital setting is attractive is the higher proportion of people from socio-economically deprived areas than in the general population, a group known to be hard to reach through screening programmes [45].

We found a lower risk of diabetes for a given glucose level in people aged over 80 years. Potential mechanisms include a healthy survivor effect, differences in glucose homeostasis, and a higher prevalence of transient causes of increased glucose in this age group such as increased use of corticosteroids and beta-agonists as a result of the higher prevalence of COPD in older people [46]. Given the high mortality rate in those aged over 80 years following hospital admission, however, many older patients with diabetes may have died without receiving a diagnosis.

We had originally intended to examine the risk of type 2 diabetes in patients aged 40 and older as type 2 diabetes is rare in patients below this age. At the review stage, however, it was noted that our cohort presents an opportunity to examine associations among younger patients, in whom the risk of type 2 diabetes is increasing. We report the findings for younger patients separately and exclude them from our risk calculator for a number of reasons: this analysis was exploratory rather than pre-specified, patients aged less than 40 are not normally offered screening for type 2 diabetes [32],[33], the overall proportion with admission glucose levels above the WHO cut-off for random glucose (11.1 mmol/l) was only around half that of patients aged 40 and older (1% versus 2%), and the subsequent risk of type 2 diabetes among those with a glucose of 11.1 mmol/l was lower (7.8% versus 9.9%) than for older patients.

Moreover, since screening is not routinely offered in people aged less than 40 years old [32],[33], and as clinicians may be less likely to diagnose type 2 diabetes as a result of its comparative rarity in this group, there may be a stronger case for routinely offering follow-up testing to patients with glucose at or above 11.1 mmol/l aged 30–39, compared to older patients, as the risk of delayed diagnosis may be higher.

Different patterns of mortality did not appear to be responsible for the weaker association between admission glucose and risk of type 2 diabetes we observed among patients admitted to ICU. Stress hyperglycaemia, first described by Claude Bernard in 1878, occurs when an acute stressor such as sepsis and other acute illness causes increased release of glucagon, cortisol, catecholamines, growth hormone, and pro-inflammatory cytokines that promote hepatic gluconeogenesis, glycogenolysis, inhibition of peripheral glucose uptake, and inhibition of insulin release [11],[12]. We suspect that for many hospitalised patients, chronically reduced beta-cell function acts in combination with these mechanisms to cause elevated glucose. Among patients acutely unwell enough to require subsequent admission to ICU, however, there may more often be a sufficiently large counter-regulatory response to produce stress hyperglycaemia in the presence of preserved pancreatic reserve. If so, this could explain the weaker association between admission glucose and subsequent diabetes in this group of patients. Nonetheless, fewer than 2,000 patients in our cohort were admitted to ICU, and there were only 37 events in this group, so this observation should be treated very cautiously.

### Limitations

One limitation of our study is that not all patients had admission glucose recorded. However, this is unlikely to have biased our estimates as the proportion with glucose measurements was high, the risk of diabetes was similar for patients who did and did not have glucose measured, and because similar results were obtained among sub-groups in whom different percentages had admission glucose measured, such as patients admitted to surgical compared to medical specialties (55% versus 80%).

The identification of incident diabetes we used was robust as all clinical care for diabetes in Scotland is delivered using the SCI-DC register from which we obtained our diagnoses. Nonetheless, our definition of type 2 diabetes relied on clinical diagnosis, so people with undiagnosed diabetes were not identified. The proportion of people with diabetes who are undiagnosed may also differ according to age, sex, or socio-economic status, which may, for example, have attenuated the strength of the association between socio-economic status and risk of type 2 diabetes. However, we had 3 and 5 years of follow-up in the main and sensitivity analyses, respectively, and would expect that the majority of patients with incipient or undiagnosed diabetes during a hospitalisation would be diagnosed within this timescale.

As we did not have serial blood glucose measurements for the period between the date of discharge from hospital and the date of diagnosis with type 2 diabetes, we are unable to state what proportion of patients with high admission glucose who were later diagnosed with type 2 diabetes had previously undetected type 2 diabetes, and what proportion experienced a temporary return to normoglycaemia, in whom the high admission glucose could be defined as isolated stress hyperglycaemia.

We did not have measures of body mass index (BMI), which may have improved the discrimination of our model. Future studies, perhaps including primary care data, in which BMI may have been recorded, are needed. In a previous study in patients with stress hyperglycaemia, however, BMI was not associated with risk of type 2 diabetes on 6-week follow-up oral glucose tolerance testing [27].

We are unable to comment on how these risks may differ by ethnicity as recording of ethnicity in hospital data for Scotland for the time period of the study was poor. Moreover, our risk calculator has not been validated in non-white populations or populations outside of Scotland.

The date of the end of follow-up was December 2011 as no more recent research extract from SCI-DC that could be linked to other databases was available at the time of publication. In the intervening period there has been no substantial change in the incidence of type 2 diabetes [42], clinical practice guidelines, or, to our knowledge as practising clinicians working in diabetes, cardiology, intensive care, and general medicine, in relevant clinical practice.

Finally, a weakness of our study is that we were unable to state whether glucose was measured on a fasting or non-fasting sample. Similarly, as the WHO only approved HbA1c for identifying type 2 diabetes as an alternative to standard glucose measures, in 2011 [32], and this use is still not widespread in Scotland, we were unable to assess the usefulness of HbA1c for diagnosing diabetes in acutely ill populations. Our results are of relevance to clinical practice however, as clinicians discharging patients from hospital, and those responsible for after-care, are frequently required to make decisions without having this information available.

### Conclusion

The 3-year risk of type 2 diabetes was <1% for patients with a glucose of ≤5 mmol/l and increased linearly to approximately 15% at 15 mmol/l, above which there was no further increase in risk. The 3-year risks at the WHO diagnostic cut-points for fasting (7 mmol/l) and random (11.1 mmol/l) glucose [34] were 3% and 10%, respectively. One in four and one in 40 patients had a glucose level above these respective cut-points. Mortality was also 1.5-fold higher in patients with glucose levels of 11.1 to 15 mmol/l compared to those with glucose levels <6.1 mmol/l. These findings can be used to inform individual patients of their long-term risk of type 2 diabetes and to offer lifestyle advice as appropriate.

## Supporting Information

Figure S1 **3-year risk of type 2 diabetes by glucose for patients in sub-groups, obtained via stratification.** Predicted 3-year risks of type 2 diabetes by glucose level obtained from logistic regression models. All models adjust for age, sex, and were stratified on the relevant grouping variable (e.g., admission to ICU). Lines represent estimates and ribbons indicate 95% CIs with blue used to indicate membership of the relevant sub-group and red used to describe the remainder of the population.
(TIFF)

Table S1 **Search terms used in brief systematic review.**
(DOCX)

Table S2 **Diabetes according to admission glucose among patients admitted to ICU.** Reports HRs from Cox regression models and sdHRs from Fine and Gray models for patients admitted to an intensive care unit.
(DOCX)

Table S3 **Comparison of patient characteristics for patients aged 30 to 39 to those in the cohort aged ≥40.**
(DOCX)

Table S4 **Baseline characteristics by admission glucose for patients aged 30 to 39.**
(DOCX)

Table S5 **Predicted risk and 95% CIs from model 1 presented in** **Table 4** **, by age, sex, and glucose.**
(XLSX)

Table S6 **Variance covariance matrix for model 1 presented in** **Table 4** **.**
(DOCX)

Table S7 **Results of brief systematic review.**
(DOCX)

Checklist S1 **STrengthening the Reporting of OBservational studies in Epidemiology (STROBE) checklist.**
(DOCX)

Protocol S1 **Statistical analysis plan.**
(PDF)

## Abbreviations

COPD: chronic obstructive pulmonary disease
csHR: cause-specific hazard ratio
HR: hazard ratio
ICU: intensive care unit
MI: myocardial infarction
OR: odds ratio
SCI-DC: Scottish Care Information-Diabetes Collaboration
sdHR: sub-distributional hazard ratio
SIMD: Scottish Index of Multiple Deprivation

## References

[1] Capes SE, Hunt D, Malmberg K, Gerstein HC (2000) Stress hyperglycaemia and increased risk of death after myocardial infarction in patients with and without diabetes: a systematic overview. Lancet 355: 773–778. 10711923 10.1016/S0140-6736(99)08415-9

[2] Kosiborod M, Rathore SS, Inzucchi SE, Masoudi FA, Wang Y, et al (2005) Admission glucose and mortality in elderly patients hospitalized with acute myocardial infarction: implications for patients with and without recognized diabetes. Circulation 111: 3078–3086. 15939812 10.1161/CIRCULATIONAHA.104.517839

[3] Capes SE, Hunt D, Malmberg K, Pathak P, Gerstein HC (2001) Stress hyperglycemia and prognosis of stroke in nondiabetic and diabetic patients: a systematic overview. Stroke J Cereb Circ 32: 2426–2432. 10.1161/hs1001.096194 11588337

[4] McAlister FA, Majumdar SR, Blitz S, Rowe BH, Romney J, et al (2005) The relation between hyperglycemia and outcomes in 2,471 patients admitted to the hospital with community-acquired pneumonia. Diabetes Care 28: 810–815. 15793178 10.2337/diacare.28.4.810

[5] Baker EH, Janaway CH, Philips BJ, Brennan AL, Baines DL, et al (2006) Hyperglycaemia is associated with poor outcomes in patients admitted to hospital with acute exacerbations of chronic obstructive pulmonary disease. Thorax 61: 284–289. 16449265 10.1136/thx.2005.051029 PMC2104606

[6] Chakrabarti B, Angus RM, Agarwal S, Lane S, Calverley PMA (2009) Hyperglycaemia as a predictor of outcome during non-invasive ventilation in decompensated COPD. Thorax 64: 857–862. 19454410 10.1136/thx.2008.106989

[7] Sleiman I, Morandi A, Sabatini T, Ranhoff A, Ricci A, et al (2008) Hyperglycemia as a predictor of in-hospital mortality in elderly patients without diabetes mellitus admitted to a sub-intensive care unit. J Am Geriatr Soc 56: 1106–1110. 18482306 10.1111/j.1532-5415.2008.01729.x

[8] Umpierrez GE, Isaacs SD, Bazargan N, You X, Thaler LM, et al (2002) Hyperglycemia: an independent marker of in-hospital mortality in patients with undiagnosed diabetes. J Clin Endocrinol Metab 87: 978–982. 11889147 10.1210/jcem.87.3.8341

[9] Gündogdu AS, Juul S, Brown PM, Sachs L, Sönksen PH (1979) Comparison of hormonal and metabolic effects of salbutamol infusion in normal subjects and insulin-requiring diabetics. Lancet 314: 1317–1321. 92670 10.1016/s0140-6736(79)92810-1

[10] Pandit MK, Burke J, Gustafson AB, Minocha A, Peiris AN (1993) Drug-induced disorders of glucose tolerance. Ann Intern Med 118: 529–539. 8442624 10.7326/0003-4819-118-7-199304010-00008

[11] Mizock BA (2001) Alterations in fuel metabolism in critical illness: hyperglycaemia. Best Pract Res Clin Endocrinol Metab 15: 533–551. 11800522 10.1053/beem.2001.0168

[12] Dungan KM, Braithwaite SS, Preiser J-C (2009) Stress hyperglycaemia. Lancet 373: 1798–1807. 19465235 10.1016/S0140-6736(09)60553-5 PMC3144755

[13] Bellamy L, Casas J-P, Hingorani AD, Williams D (2013) Type 2 diabetes mellitus after gestational diabetes: a systematic review and meta-analysis. Lancet 373: 1773–1779. 19465232 10.1016/S0140-6736(09)60731-5

[14] Petursson P, Herlitz J, Caidahl K, From-Attebring M, Sjöland H, et al (2006) Association between glycometabolic status in the acute phase and 2½ years after an acute coronary syndrome. Scand Cardiovasc J 40: 145–151. 16798661 10.1080/14017430600797626

[15] Lankisch M, Füth R, Gülker H, Lapp H, Bufe A, et al (2008) Screening for undiagnosed diabetes in patients with acute myocardial infarction. Clin Res Cardiol 97: 753–759. 18491170 10.1007/s00392-008-0674-5

[16] Knudsen EC, Seljeflot I, Abdelnoor M, Eritsland J, Mangschau A, et al (2009) Abnormal glucose regulation in patients with acute ST- elevation myocardial infarction-a cohort study on 224 patients. Cardiovasc Diabetol 8: 6. 19183453 10.1186/1475-2840-8-6 PMC2646717

[17] Meisinger C, Beck J, Heier M, Hörmann A, Kuch B, et al (2010) Myocardial infarction and incidence of type 2 diabetes mellitus. Is admission blood glucose an independent predictor for future type 2 diabetes mellitus? Am Heart J 159: 258–263. 20152224 10.1016/j.ahj.2009.11.027

[18] Gray CS, Scott JF, French JM, Alberti KGMM, O'Connell JE (2004) Prevalence and prediction of unrecognised diabetes mellitus and impaired glucose tolerance following acute stroke. Age Ageing 33: 71–77. 14695867 10.1093/ageing/afh026

[19] Jia Q, Zheng H, Liu L, Zhao X, Wang C, et al (2010) Persistence and predictors of abnormal glucose metabolisms in patients after acute stroke. Neurol Res 32: 359–365. 20483001 10.1179/016164110X12656393665242

[20] MacIntyre EJ, Majumdar SR, Gamble J-M, Minhas-Sandhu JK, Marrie TJ, et al (2012) Stress hyperglycemia and newly diagnosed diabetes in 2124 patients hospitalized with pneumonia. Am J Med 125: 1036.e17–e23. 22863217 10.1016/j.amjmed.2012.01.026

[21] Oswald GA, Yudkin JS (1987) Hyperglycaemia following acute myocardial infarction: the contribution of undiagnosed diabetes. Diabet Med J Br Diabet Assoc 4: 68–70. 10.1111/j.1464-5491.1987.tb00833.x 2951225

[22] Norhammar A, Tenerz A, Nilsson G, Hamsten A, Efendíc S, et al (2002) Glucose metabolism in patients with acute myocardial infarction and no previous diagnosis of diabetes mellitus: a prospective study. Lancet 359: 2140–2144. 12090978 10.1016/S0140-6736(02)09089-X

[23] Wahid ST, Sultan J, Handley G, Saeed BO, Weaver JU, et al (2002) Serum fructosamine as a marker of 5-year risk of developing diabetes mellitus in patients exhibiting stress hyperglycaemia. Diabet Med J Br Diabet Assoc 19: 543–548. 10.1046/j.1464-5491.2002.00730.x 12099956

[24] George PM, Valabhji J, Dawood M, Henry JA (2005) Screening for type 2 diabetes in the accident and emergency department. Diabet Med J Br Diabet Assoc 22: 1766–1769. 10.1111/j.1464-5491.2005.01674.x 16401327

[25] Ishihara M, Inoue I, Kawagoe T, Shimatani Y, Kurisu S, et al (2006) Is admission hyperglycaemia in non-diabetic patients with acute myocardial infarction a surrogate for previously undiagnosed abnormal glucose tolerance? Eur Heart J 27: 2413–2419. 17000629 10.1093/eurheartj/ehl271

[26] Okosieme OE, Peter R, Usman M, Bolusani H, Suruliram P, et al (2008) Can admission and fasting glucose reliably identify undiagnosed diabetes in patients with acute coronary syndrome? Diabetes Care 31: 1955–1959. 18591399 10.2337/dc08-0197 PMC2551634

[27] Charfen MA, Ipp E, Kaji AH, Saleh T, Qazi MF, et al (2009) Detection of undiagnosed diabetes and prediabetic states in high-risk emergency department patients. Acad Emerg Med Off J Soc Acad Emerg Med 16: 394–402. 10.1111/j.1553-2712.2009.00374.x 19302369

[28] Dave JA, Engel ME, Freercks R, Peter J, May W, et al (2010) Abnormal glucose metabolism in non-diabetic patients presenting with an acute stroke: prospective study and systematic review. QJM 103: 495–503. 20430755 10.1093/qjmed/hcq062

[29] Witte DR, Shipley MJ, Marmot MG, Brunner EJ (2010) Performance of existing risk scores in screening for undiagnosed diabetes: an external validation study. Diabet Med 27: 46–53. 20121888 10.1111/j.1464-5491.2009.02891.x

[30] Rathmann W, Martin S, Haastert B, Icks A, Holle R, et al (2005) Performance of screening questionnaires and risk scores for undiagnosed diabetes: the kora survey 2000. Arch Intern Med 165: 436–441. 15738374 10.1001/archinte.165.4.436

[31] Anwar H, Fischbacher CM, Leese GP, Lindsay RS, McKnight JA, et al (2011) Assessment of the under-reporting of diabetes in hospital admission data: a study from the Scottish Diabetes Research Network Epidemiology Group: under-reporting of diabetes in hospital admission data. Diabet Med 28: 1514–1519. 21883441 10.1111/j.1464-5491.2011.03432.x PMC4215191

[32] NICE (2012) PH38 Preventing type 2 diabetes - risk identification and interventions for individuals at high risk: guidance. Available: http://publications.nice.org.uk/preventing-type-2-diabetes-risk-identification-and-interventions-for-individuals-at-high-risk-ph38. Accessed 25 April 2014.

[33] American Diabetes Association (2012) Standards of Medical Care in Diabetes–2013. Diabetes Care 36: S11–S66. 10.2337/dc13-S011 PMC3537269 23264422

[34] WHO | Definition and diagnosis of diabetes mellitus and intermediate hyperglycaemia (n.d.). WHO. Available: http://www.who.int/diabetes/publications/diagnosis_diabetes2006/en/index.html. Accessed 27 September 2013.

[35] Fine JP, Gray RJ (1999) A proportional hazards model for the subdistribution of a competing risk. J Am Stat Assoc 94: 496–509.

[36] Therneau TM (2014) A Package for Survival Analysis in S. Available: http://CRAN.R-project.org/package=survival.

[37] Gray B (2013) cmprsk: Subdistribution Analysis of Competing Risks. Available: http://CRAN.R-project.org/package=cmprsk.

[38] Jelinek GA, Weiland TJ, Moore G, Tan G, Maslin M, et al (2010) Screening for type 2 diabetes with random finger-prick glucose and bedside HbA1c in an Australian emergency department. Emerg Med Australas 22: 427–434. 21040483 10.1111/j.1742-6723.2010.01333.x

[39] Gornik I, Vujaklija-Brajkovic A, Renar IP, Gasparovic V (2010) A prospective observational study of the relationship of critical illness associated hyperglycaemia in medical ICU patients and subsequent development of type 2 diabetes. Crit Care 14: R130. 20615210 10.1186/cc9101 PMC2945097

[40] Gornik I, Vujaklija A, Lukić E, Madžarac G, Gašparović V (2010) Hyperglycaemia in critical illness is a risk factor for later development of type II diabetes mellitus. Acta Diabetol 47: 29–33. 19340390 10.1007/s00592-009-0115-6

[41] National Institute for Health and Clinical Excellence (2011) Hyperglycaemia in acute coronary syndromes. Management of hyperglycaemia in acute coronary syndromes. NICE Clinical Guideline Available: http://www.nice.org.uk/. Accessed 8 March 2014.

[42] Scottish Diabetes Survey Monitoring Group (2014) Scottish Diabetes Survey 2013. Diabetes Scotl Available: http://www.diabetesinscotland.org.uk/Publications.aspx. Accessed 6 June 2014.

[43] Waugh N, Shyangdan D, Taylor-Phillips S, Suri G, Hall B (2013) Screening for type 2 diabetes: a short report for the National Screening Committee. Health Technol Assess Winch Engl 17: 1–90. 10.3310/hta17350 PMC4780946 23972041

[44] Rydén L, Grant PJ, Anker SD, Berne C, Cosentino F, et al (2013) ESC Guidelines on diabetes, pre-diabetes, and cardiovascular diseases developed in collaboration with the EASD The Task Force on diabetes, pre-diabetes, and cardiovascular diseases of the European Society of Cardiology (ESC) and developed in collaboration with the European Association for the Study of Diabetes (EASD). Eur Heart J eht108. 10.1093/eurheartj/eht108 23996285

[45] Maheswaran R, Pearson T, Jordan H, Black D (2006) Socioeconomic deprivation, travel distance, location of service, and uptake of breast cancer screening in North Derbyshire, UK. J Epidemiol Community Health 60: 208–212. 16476749 10.1136/jech.200X.038398 PMC2465550

[46] Buist AS, McBurnie MA, Vollmer WM, Gillespie S, Burney P, et al (2007) International variation in the prevalence of COPD (The BOLD Study): a population-based prevalence study. Lancet 370: 741–750. 17765523 10.1016/S0140-6736(07)61377-4

