# Supplementary figures and images for: Stress Hyperglycaemia in Hospitalised Patients and Their 3-Year Risk of Diabetes: A Scottish Retrospective Cohort Study
Source: PLoS Med. 2014 Aug 19;11(8):e1001708. doi: 10.1371/journal.pmed.1001708 (PMC4138030; doi:10.1371/journal.pmed.1001708)

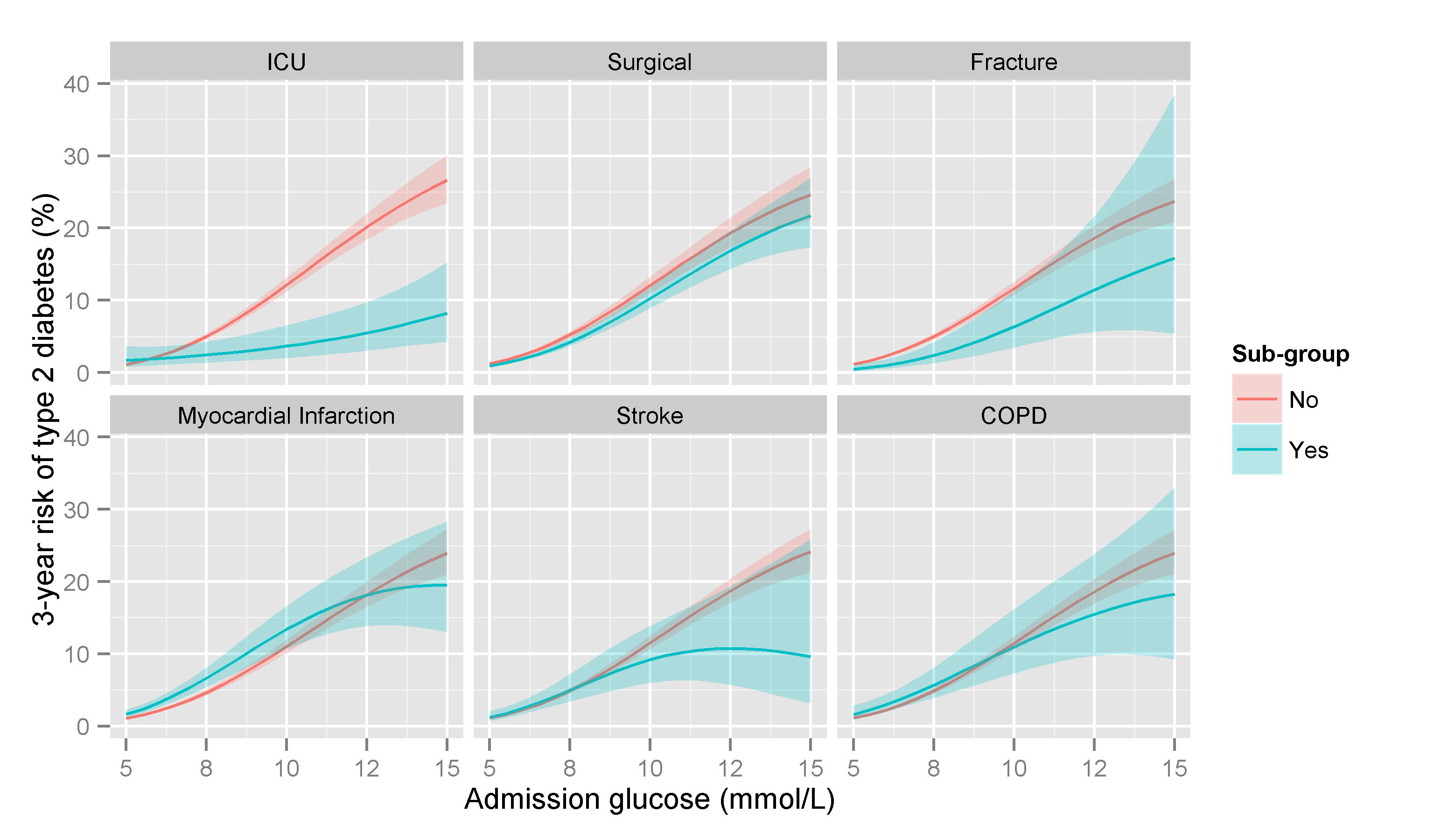

Supplement: Figure S1 — 3-year risk of type 2 diabetes by glucose for patients in sub-groups, obtained via stratification. Predicted 3-year risks of type 2 diabetes by glucose level obtained from logistic regression models. All models adjust for age, sex, and were stratified on the relevant grouping variable (e.g., admission to ICU). Lines represent estimates and ribbons indicate 95% CIs with blue used to indicate membership of the relevant sub-group and red used to describe the remainder of the population. (TIFF) [file pmed.1001708.s001.tiff]
