# Supplementary material for: Stress Hyperglycaemia in Hospitalised Patients and Their 3-Year Risk of Diabetes: A Scottish Retrospective Cohort Study
Source: PLoS Med. 2014 Aug 19;11(8):e1001708. doi: 10.1371/journal.pmed.1001708 (PMC4138030; doi:10.1371/journal.pmed.1001708)
Supplement: Table S1 — Search terms used in brief systematic review. (DOCX) [file pmed.1001708.s002.docx]

Table S1 Search strategy

Database Medline

Date14th of October 2013

| 1 | acute disease/ or critical illness/ or emergencies/ |
| --- | --- |
| 2 | admitting department, hospital/ or emergency service, hospital/ |
| 3 | exp Myocardial Ischemia/ |
| 4 | exp Stroke/ |
| 5 | exp Pulmonary Disease, Chronic Obstructive/ |
| 6 | Blood Glucose/ |
| 7 | Hyperglycemia/ |
| 8 | Glucose.mp |
| 9 | hypergly.mp |
| 10 | stress hypergly*.mp. [mp=title, abstract, original title, name of substance word, subject heading word, keyword heading word, protocol supplementary concept, rare disease supplementary concept, unique identifier] |
| 11 | exp Cohort Studies/ |
| 12 | exp incidence/ or exp prevalence/ |
| 13 | exp Mass screening/ |
| 14 | humans/ |
|  | ((1 or 2 or 3 or 4 or 5) and (6 or 7 or 8 or 9)) or 6 and ((11 or 12 or 13) and 14) - limit to yr="2000-Current. |
