# Supplementary material for: Stress Hyperglycaemia in Hospitalised Patients and Their 3-Year Risk of Diabetes: A Scottish Retrospective Cohort Study
Source: PLoS Med. 2014 Aug 19;11(8):e1001708. doi: 10.1371/journal.pmed.1001708 (PMC4138030; doi:10.1371/journal.pmed.1001708)
Supplement: Table S2 — Diabetes according to admission glucose among patients admitted to ICU. Reports HRs from Cox regression models and sdHRs from Fine and Gray models for patients admitted to an intensive care unit. (DOCX) [file pmed.1001708.s003.docx]

Table S2. Diabetes according to admission glucose among patients admitted to ICU. Reports cause-specific hazard ratios from Cox regression models and sub-distribution hazard ratios from Fine and Gray models for patients admitted to an intensive care unit.

|  | Cox regression | Fine and Gray regression |
| --- | --- | --- |
| Age, per ten years | 39.74; 95%CI (2.17 to 726.97) | 49.11; 95%CI (2.54 to 950.90) |
| Age, per ten years, squared | 0.76; 95%CI (0.61 to 0.95) | 0.74; 95%CI (0.59 to 0.93) |
| Male | 1.23; 95%CI (0.69 to 2.17) | 1.19; 95%CI (0.67 to 2.10) |
| Glucose, mmol/L |  |  |
| ≤ 7 | 1 | 1 |
| 7 to 11.1 | 1.62; 95%CI (0.83 to 3.16) | 1.59; 95%CI (0.81 to 3.11) |
| > 11.1 | 3.39; 95%CI (1.62 to 7.06) | 3.51; 95%CI (1.67 to 7.36) |

csHR – Cause-specific hazard ratio (95% confidence interval). sdHR – sub-distribution hazard ratio (95% confidence interval)All models adjust for age, sex and glucose.

N= 1828, number of diabetes events = 48
