# Supplementary material for: Stress Hyperglycaemia in Hospitalised Patients and Their 3-Year Risk of Diabetes: A Scottish Retrospective Cohort Study
Source: PLoS Med. 2014 Aug 19;11(8):e1001708. doi: 10.1371/journal.pmed.1001708 (PMC4138030; doi:10.1371/journal.pmed.1001708)
Supplement: Table S3 — Comparison of patient characteristics for patients aged 30 to 39 to those in the cohort aged ≥40. (DOCX) [file pmed.1001708.s004.docx]

Table S3. Comparison of patient characteristics for patients aged 30 to 39 to those in the cohort aged ≥ 40.

|  | Age ≥ 40 | Age 30 to 39 |
| --- | --- | --- |
| N | 86,512 | 11,875 |
| Age, years | 66.3 (15.1) | 35 (2.9) |
| Male | 40,596 (46.9) | 6,053 (51.0) |
| SIMD quintile |  |  |
| Q1 (most deprived) | 24,962 (28.9) | 4,071 (34.3) |
| Q2 | 18,786 (21.7) | 2,600 (21.9) |
| Q3 | 13,207 (15.3) | 1,849 (15.6) |
| Q4 | 12,087 (14) | 1,603 (13.5) |
| Q5 (least deprived) | 17,,470 (20.2) | 1,752 (14.8) |
| WCC>11 | 27,448 (31.7) | 10,647 (89.7) |
| Charlson index |  |  |
| 0 | 54,770 (63.3) | 10,477 (88.2) |
| 1 to 4 | 7,058 (8.2) | 70 (0.6) |
| 5 to 6 | 4,188 (4.8) | 701 (5.9) |
| > 6 | 20,496 (23.7) | 110 (0.9) |
| Medical specialty | 61,153 (70.7) | 6,594 (55.5) |
| Intensive Care Unit | 1,828 (2.1) | 247 (2.1) |
| Myocardial infarction | 6,204 (7.17) | 120 (1.0) |
| Stroke | 3,723 (4.3) | 79 (0.7) |
| Chronic Obstructive Pulmonary Disease | 3,003 (3.47) | 21 (0.2) |
| Fracture | 4,976 (5.75) | 343 (2.9) |

Number (percentage of patients with characteristic for patients aged 30 to 39 compared to the original cohort
