# Supplementary material for: Stress Hyperglycaemia in Hospitalised Patients and Their 3-Year Risk of Diabetes: A Scottish Retrospective Cohort Study
Source: PLoS Med. 2014 Aug 19;11(8):e1001708. doi: 10.1371/journal.pmed.1001708 (PMC4138030; doi:10.1371/journal.pmed.1001708)
Supplement: Table S4 — Baseline characteristics by admission glucose for patients aged 30 to 39. (DOCX) [file pmed.1001708.s005.docx]

Table S4 Baseline characteristics by admission glucose for patients aged 30 to 39

| Glucose (mmol/L) | <6.1 | 6.1-6.9 | 7.0-11.0 | 11.1-14.9 | ≥15 |
| --- | --- | --- | --- | --- | --- |
| N | 8451 | 1866 | 1438 | 98 | 22 |
| Age, years | 34.8 (2.9) | 34.8 (2.9) | 34.9 (2.8) | 35.1 (2.5) | 34.7 (3.0) |
| Male | 4561 (54.0%) | 836 (44.8%) | 611 (42.5%) | 36 (36.7%) | 9 (40.9%) |
| SIMD quintile |  |  |  |  |  |
| Q1 (most deprived) | 2893 (34.2) | 620 (33.2) | 516 (35.9) | 37 (37.8) | 5 (22.7) |
| Q2 | 1855 (22.0) | 401 (21.5) | 319 (22.2) | 23 (23.5) | 2 (9.1) |
| Q3 | 1302 (15.4) | 308 (16.5) | 219 (15.2) | 15 (15.3) | 5 (22.7) |
| Q4 | 1151 (13.6) | 262 (14.0) | 175 (12.2) | 11 (11.2) | 4 (18.2) |
| Q5 (least deprived) | 1250 (14.8) | 275 (14.7) | 209 (14.5) | 12 (12.2) | 6 (27.3) |
| WCC>11 | 7590 (89.8) | 1661 (89.0) | 1286 (89.4) | 89 (90.8) | 21 (95.5) |
| Charlson index 0 | 7517 (88.9) | 1635 (87.6) | 1227 (85.3) | 80 (81.6) | 18 (81.8) |
| Medical specialty | 4612 (54.6) | 1054 (56.5) | 843 (58.6) | 67 (68.4) | 18 (81.8) |
| Intensive Care Unit | 108 (1.3) | 42 (2.3) | 78 (5.4) | 12 (12.2) | 7 (31.8) |
| Myocardial infarction | 67 (0.8) | 20 (1.1) | 29 (2.0) | 3 (3.1) | 1 (4.5) |
| Stroke | 42 ( 0.5) | 19 ( 1.0) | 16 ( 1.1) | 2 ( 2.0) | 0 ( 0.0) |
| Chronic Obstructive Pulmonary Disease | 13 ( 0.2) | 2 ( 0.1) | 6 ( 0.4) | 0 ( 0.0) | 0 ( 0.0) |
| Fracture | 210 (2.5) | 77 (4.1) | 50 (3.5) | 5 (5.1) | 1 (4.5) |

All data are n (%) except age which is reported as mean (standard deviation).
SIMD – Scottish Index of Multiple Deprivation
