# Supplementary material for: Stress Hyperglycaemia in Hospitalised Patients and Their 3-Year Risk of Diabetes: A Scottish Retrospective Cohort Study
Source: PLoS Med. 2014 Aug 19;11(8):e1001708. doi: 10.1371/journal.pmed.1001708 (PMC4138030; doi:10.1371/journal.pmed.1001708)
Supplement: Table S6 — Variance covariance matrix for model 1 presented in Table 4 . (DOCX) [file pmed.1001708.s007.docx]

Table S6. Variance covariance matrix for model 1 presented in Table 4

|  | Intercept | Age (ten years) | Age (ten years) squared | Sex, male | Log-glucose | Log-glucose squared | Log-glucose cubed |
| --- | --- | --- | --- | --- | --- | --- | --- |
| Intercept | 3.54E-01 | -9.58E-02 | 7.34E-03 | -2.32E-03 | 1.58E-02 | -4.92E-02 | 1.38E-02 |
| Age (ten years) | -9.58E-02 | 3.12E-02 | -2.41E-03 | 4.68E-04 | 6.68E-04 | -1.53E-03 | 3.87E-04 |
| Age (ten years) squared | 7.34E-03 | -2.41E-03 | 1.88E-04 | -4.53E-05 | -3.40E-05 | 8.19E-05 | -2.09E-05 |
| Sex, male | -2.32E-03 | 4.68E-04 | -4.53E-05 | 2.24E-03 | -1.17E-04 | 2.40E-04 | -5.86E-05 |
| Log-glucose | 1.58E-02 | 6.68E-04 | -3.40E-05 | -1.17E-04 | 2.69E-02 | -3.61E-02 | 8.88E-03 |
| Log-glucose squared | -4.92E-02 | -1.53E-03 | 8.19E-05 | 2.40E-04 | -3.61E-02 | 6.99E-02 | -1.86E-02 |
| Log-glucose cubed | 1.38E-02 | 3.87E-04 | -2.09E-05 | -5.86E-05 | 8.88E-03 | -1.86E-02 | 5.03E-03 |
