# Supplementary material for: Stress Hyperglycaemia in Hospitalised Patients and Their 3-Year Risk of Diabetes: A Scottish Retrospective Cohort Study
Source: PLoS Med. 2014 Aug 19;11(8):e1001708. doi: 10.1371/journal.pmed.1001708 (PMC4138030; doi:10.1371/journal.pmed.1001708)
Supplement: Table S7 — Results of brief systematic review. (DOCX) [file pmed.1001708.s008.docx]

Table S7 Results of Systematic review

# Study characteristics

| First Author | Publication year | Recruitment | Country | Included specific diseases | Centre | Department | Inclusion criteria | Exclusion criteria | Sample method |
| --- | --- | --- | --- | --- | --- | --- | --- | --- | --- |
| Wahid ST[1] | 2002 | 1994-1995 | Australia | No | Single | Emergency Department | Aged >16 and had venesection in ED and random serum glucose ≥11.1mmol/L | None | Unclear |
| Gray CS[2] | 2004 | 1997-1999 | UK | Stroke | Single | Admissions Unit | Adults with stroke, admission plasma glucose between 6 and 17 mmol/l | None | Pragmatic randomised controlled trial. Included 142 of 398 eligible |
| George PM[3] | 2005 | 2003 | UK | No | Single | Emergency Department | Aged >40 | None | Consecutive sample. Included 500 of 570 eligible |
| Petursson P[4] | 2006 | 1995-1999 | Sweden | Acute myocardial infarction | Single | Coronary Care Unit | Acute myocardial infarction, chest pain or possible myocardial ischaemia | Known diabetes | Unclear |
| Lankisch M[5] | 2008 | 2003-2004 | Germany | Acute myocardial infarction | Single | Tertiary Centre | Adults | None | Consecutive sample |
| Knudsen EC[6] | 2009 | 2005-2007 | Norway | ST-elevation myocardial infarction treated via thrombolysis | Single | Coronary Care Unit | Adults | Known diabetes or impaired glucose tolerance | Unclear |
| Charfen MA[7] | 2009 | 2004-2006 | USA | No | Single | Emergency Department | Aged 18 to 80 years, random serum glucose ≥140 mg/dl (7.8 mmol/L) or ≥ 126 mg/dl (7 mmol/L) if 2 hours since food or if had ADA risk factors or were admitted with a soft tissue infection | Known diabetes, or diabetes diagnosed unequivocally in emergency department, glucocorticosteroid use or severe acute illness | Convenience sample |
| Jelinek GA[8] | 2010 | 2007-2008 | Australia | No | Single | Emergency Department | Aged ≥18 years, attending ED | Unable to consent, pregnant, very acutely unwell, treated with intravenous glucose | Convenience sample |
| Jia Q[9] | 2010 | 2009 | China | Stroke | Single | Neurology Unit | Aged >18, first stroke | Known diabetes | Consecutive sample |
| Gornik I [10](Critical Care) | 2010 | 1998-2004 | Croatia | No | Single | Medical Intensive Care Unit | Adults | Known diabetes, impaired fasting glucose, impaired glucose tolerance, any other endocrine disorder, or corticosteroid therapy | Consecutive sample, 1105 of 1316 |
| Gornik I [11](Acta Diabetelogia) | 2010 | 2000-2002 | Croatia | Sepsis, acute coronary syndrome and/or heart failure | Single | Medical Intensive Care Unit | Adults | Known diabetes, died in hospital, corticosteroid therapy, endocrine disorder affecting glucose metabolism, any condition thought likely to hinder follow-up | Consecutive sample, 331 of 390 eligible |
| Meisinger C[12] | 2010 | 1998-2003 | Germany | Acute myocardial infarction | Multi-centre | Regional Coronary Event Registry | Adults | None | Disease register |
| MacIntyre EJ[13] | 2012 | 2000-2002 | Canada | Community acquired pneumonia | Multi-centre | Six Hospitals | Aged >17 | Self-reported diabetes, diabetes medication, diabetes meal plan, died in hospital | Consecutive sample |

# Definitions and covariates

| First Author | Recruited | Age | Female | Exposure | Outcome | Covariates |
| --- | --- | --- | --- | --- | --- | --- |
| Wahid ST[1] | 317 | 59.7 | 0.43 | Random serum glucose ≥11.1mmol/L | Electronic and manual searches of routine data (A&E notes, biochemistry database, local diabetes register, GP records) from 1999-2000 | None |
| Gray CS[2] | 142∙ | 75 | 0.47 | Admission plasma glucose ≥6.1 mmol/L | OGTT at 12 weeks, fasting blood glucose ≥7 or 2hr ≥11.1 mmol/L | None |
| George PM[3] | 500 | Not reported | Not reported | Random capillary blood glucose > 7 mmol/L | Fasting blood glucose ≥7 mmol/L (on 2 occasions or on 1 occasion if had osmotic symptoms) | None |
| Petursson P[4] | 762 | 65 (median) | 0.28 | Random plasma glucose on admission, categorised as <6.1, 6.1-6.9 and ≥7 mmol/L, n = 324, 184 and 254 respectively | Self-reported status or fasting plasma glucose ≥7 mmol/L at 2.5 years follow-up | None |
| Lankisch M[5] | 96 | 62.7 | 0.28 | Random plasma glucose on admission (no cut-offs) | OGTT at 12 weeks | None |
| Knudsen EC[6] | 224 | 58 | 0.17 | Post procedure plasma glucose ≥7.7 | OGTT at 12 weeks, fasting plasma glucose ≥7 or 2hr ≥11.1 mmol/L | Age |
| Charfen MA[7] | 528 | 48.7 | 0.45 | As per inclusion criteria | A combination of criteria according to fasting capillary blood glucose, fasting plasma glucose and OGTT at 6-weeks, which were repeated on a second occasion for patients who did not have an unequivocal diagnosis on a single occasion (fasting plasma glucose >150 mg/dL and HbA1c >8%) | Age, sex and body mass index |
| Jelinek GA[8] | 725 | 56 | 0.5 | Random capillary blood glucose >6 mmol/L or HbA1c>6.0% | OGTT at ≥ 1 week | None |
| Jia Q[9] | 123 | 59.1 | 0.36 | Per 1 mmol/l of glucose measured fasting on day after admission | OGTT at 12 weeks, fasting plasma glucose ≥7 or 2hr ≥11.1 | History of hypertension, fasting plasma glucose and 2 hour post load glucose on day 14 following admission |
| Gornik I [10](Critical Care) | 1105 | 58 | 0.45 | Random blood glucose >7.7mmol/L | OGTT at 6 weeks then annually | None |
| Gornik I [11](Acta Diabetelogia) | 258 | 57.9 | 0.46 | 2 or more random blood glucose ≥ 7.8 mmol/L | Not described | None |
| Meisinger C[12] | 1239 | 33.8% aged 65-74 | 0.24 | First serum glucose, quartiles | Follow-up questionnaires, validated by hospital record or physician interview | Age, sex, treatment with β-blocker, diuretics, history of hypertension, hyperlipidaemia, smoking, and body mass index. |
| MacIntyre EJ[13] | 2215 | 68 | 0.49 | Random serum glucose, categorised 4-6mmol/l (normal), 6.1-7.7 mmol/l (mild), 7.8-11.0 mmol/L (mod), 11.1-20.0mmol/L (severe) | New diagnosis within 5 years identified via administrative data | Age, sex, history of cardiovascular disease, neoplasm, or chronic obstructive pulmonary disease, smoking status, total number of prescription medications, use of statins, nursing home residence, functional status, and a pneumonia-specific severity index |

# Follow-up and findings

| First Author | Follow-up | Completeness of follow-up (%) | Statistical analysis | Finding |
| --- | --- | --- | --- | --- |
| Wahid ST[1] | 5 years | 70.7 | Simple proportion | 63/224 (28%) had diabetes at follow-up |
| Gray CS[2] | 12 weeks | 43.7 | Simple proportion | 14/41 (34.1%) had diabetes at follow-up |
| George PM[3] | ≥ 1 week | 100 | Simple proportion | 13/36 (36.1%) had diabetes at follow-up |
| Petursson P[4] | 2.5 years | Unclear | Simple proportion | Overall estimate not provided. Stratified by admission glucose categories the percentage with diabetes was: <6.1 mmol/L 5%, 6.1-6.9 mmol/L 11%, and ≥7 mmol/L 24% |
| Lankisch M[5] | 12 weeks | 64.6 | Pearson correlation coefficient | Correlation coefficient (r) was 0.45 for admission and follow-up plasma glucose |
| Knudsen EC[6] | 12 weeks | 89.3 | Logistic regression | Adjusted OR 2.59 (1.31-5.12) |
| Charfen MA[7] | 6 weeks | 48.1 | Logistic regression | 27/256 (10.5%) patients had diabetes at 6 weeks. For those with random serum glucose ≥140 mg/dL, 36/246 (14.6%) had diabetes at 6 weeks. OR 1.36 (95%CI 1.15 to 1.61) per 10mg/dl glucose (adjusting for HbA1c and glucose) age, sex and body mass index were not associated with risk of diabetes |
| Jelinek GA[8] | ≥1 week | 23.4 | Simple proportion | 6/45 (13.3%) had diabetes at follow-up |
| Jia Q[9] | 12 weeks | 87 | Logistic regression | Adjusted OR 4.94 (95% CI 1.24-20.0) per 1 mmol/L increment in admission fasting glucose |
| Gornik I [10](Critical Care) | 5 years | 52.7 | Simple proportion | 76/436 (17.4%) had diabetes at 6 weeks, 47/193 (24.4%) had diabetes at 5 years (the latter group excluded people who had diabetes at 6 weeks) |
| Gornik I [11](Acta Diabetelogia) | 5 years | 54.8 | Simple proportion | 8/51 (15.7%) with hyperglycaemia had diabetes at follow-up |
| Meisinger C[12] | Mean follow-up 4.7 years | Not reported | Cox regression | Hazard ratio (HR) 2.76 for age and sex and 2.59 adjusting for all covariates |
| MacIntyre EJ[13] | 5 years | 95.9 | Simple proportion with out of hospital deaths retained in denominator, Cox regression treating death as an uninformative censoring event, and Fine and Gray model for competing risks treating death as a competing event. | Overall estimate not provided. Stratified by admission glucose categories the percentage with diabetes was: Normal 45/706 (6%), mild 59/841 (7%), moderate 86/473 (18%) and severe 49 of 104 (47%) |

OGTT –oral glucose tolerance test

OR – odds ratio

References

1. Wahid ST, Sultan J, Handley G, Saeed BO, Weaver JU, et al. (2002) Serum fructosamine as a marker of 5-year risk of developing diabetes mellitus in patients exhibiting stress hyperglycaemia. Diabet Med J Br Diabet Assoc 19: 543–548.

2. Gray CS, Scott JF, French JM, Alberti KGMM, O’Connell JE (2004) Prevalence and prediction of unrecognised diabetes mellitus and impaired glucose tolerance following acute stroke. Age Ageing 33: 71–77. doi:10.1093/ageing/afh026.

3. George PM, Valabhji J, Dawood M, Henry JA (2005) Screening for Type 2 diabetes in the accident and emergency department. Diabet Med J Br Diabet Assoc 22: 1766–1769. doi:10.1111/j.1464-5491.2005.01674.x.

4. Petursson P, Herlitz J, Caidahl K, From-Attebring M, Sjöland H, et al. (2006) Association between glycometabolic status in the acute phase and 2½ years after an acute coronary syndrome. Scand Cardiovasc J 40: 145–151. doi:10.1080/14017430600797626.

5. Lankisch M, Füth R, Gülker H, Lapp H, Bufe A, et al. (2008) Screening for undiagnosed diabetes in patients with acute myocardial infarction. Clin Res Cardiol 97: 753–759. doi:10.1007/s00392-008-0674-5.

6. Knudsen EC, Seljeflot I, Abdelnoor M, Eritsland J, Mangschau A, et al. (2009) Abnormal glucose regulation in patients with acute ST- elevation myocardial infarction-a cohort study on 224 patients. Cardiovasc Diabetol 8: 6. doi:10.1186/1475-2840-8-6.

7. Charfen MA, Ipp E, Kaji AH, Saleh T, Qazi MF, et al. (2009) Detection of undiagnosed diabetes and prediabetic states in high-risk emergency department patients. Acad Emerg Med Off J Soc Acad Emerg Med 16: 394–402. doi:10.1111/j.1553-2712.2009.00374.x.

8. Jelinek GA, Weiland TJ, Moore G, Tan G, Maslin M, et al. (2010) Screening for type 2 diabetes with random finger-prick glucose and bedside HbA1c in an Australian emergency department. Emerg Med Australas 22: 427–434. doi:10.1111/j.1742-6723.2010.01333.x.

9. Jia Q, Zheng H, Liu L, Zhao X, Wang C, et al. (2010) Persistence and predictors of abnormal glucose metabolisms in patients after acute stroke. Neurol Res 32: 359–365. doi:10.1179/016164110X12656393665242.

10. Gornik I, Vujaklija-Brajkovic A, Renar IP, Gasparovic V (2010) A prospective observational study of the relationship of critical illness associated hyperglycaemia in medical ICU patients and subsequent development of type 2 diabetes. Crit Care 14: R130. doi:10.1186/cc9101.

11. Gornik I, Vujaklija A, Lukić E, Madžarac G, Gašparović V (2010) Hyperglycaemia in critical illness is a risk factor for later development of type II diabetes mellitus. Acta Diabetol 47: 29–33. doi:10.1007/s00592-009-0115-6.

12. Meisinger C, Beck J, Heier M, Hörmann A, Kuch B, et al. (2010) Myocardial infarction and incidence of type 2 diabetes mellitus. Is admission blood glucose an independent predictor for future type 2 diabetes mellitus? Am Heart J 159: 258–263. doi:10.1016/j.ahj.2009.11.027.

13. MacIntyre EJ, Majumdar SR, Gamble J-M, Minhas-Sandhu JK, Marrie TJ, et al. (2012) Stress hyperglycemia and newly diagnosed diabetes in 2124 patients hospitalized with pneumonia. Am J Med 125: 1036.e17–23. doi:10.1016/j.amjmed.2012.01.026.
