# Supplementary material for: Stress Hyperglycaemia in Hospitalised Patients and Their 3-Year Risk of Diabetes: A Scottish Retrospective Cohort Study
Source: PLoS Med. 2014 Aug 19;11(8):e1001708. doi: 10.1371/journal.pmed.1001708 (PMC4138030; doi:10.1371/journal.pmed.1001708)
Supplement: Checklist S1 — STrengthening the Reporting of OBservational studies in Epidemiology (STROBE) checklist. (DOCX) [file pmed.1001708.s009.docx]

STROBE Statement—checklist of items that should be included in reports of observational studies

|  | Item No | Recommendation |  |
| --- | --- | --- | --- |
| **Title and abstract** | 1 | (*a*) Retrospective cohort study (page 1) |  |
|  |  | (*b*) page 2 | √ |
| Introduction | | |  |
| Background/rationale | 2 | Page 3 | √ |
| Objectives | 3 | Objective was to describe risk with precision, not to test hypothesis page 4 | √ |
| Methods | | |  |
| Study design | 4 | Pages 4 and 5 | √ |
| Setting | 5 | Setting (page 5), locations (page 5), and relevant dates (page 5, para 3), including periods of recruitment, exposure, follow-up, and data collection | √ |
| Participants | 6 | (*a*) *Cohort study*—Give the eligibility criteria, and the sources and methods of selection of participants. Describe methods of follow-up  Page 4, heading outcomes | √ |
|  |  |  |  |
| Variables | 7 | Clearly define all outcomes, exposures, predictors, potential confounders, and effect modifiers. Give diagnostic criteria, if applicable  Page 5, headings outcomes, exposures, covariates | √ |
| Data sources/ measurement | 8* | For each variable of interest, give sources of data and details of methods of assessment (measurement). Describe comparability of assessment methods if there is more than one group – as above | √ |
| Bias | 9 | Describe any efforts to address potential sources of bias- NA | √ |
| Study size | 10 | Based on precision, page 4 | √ |
| Quantitative variables | 11 | Page 5, heading statistical analysis | √ |
| Statistical methods | 12 | (*a*) Page 6, heading statistical analysis | √ |
|  |  | (*b*) Page 6, heading statistical analysis | √ |
|  |  | (*c*) See Figure 1, patients without glucose measures were not included in the analysis | √ |
|  |  | (*d*) *Cohort study*—NA – there was no loss to follow-up | √ |
|  |  | (*e*) Describe any sensitivity analyses, page 7, sensitivity analysis | √ |

Continued on next page

| Results | | |  |
| --- | --- | --- | --- |
| Participants | 13* | (a) Figure 1, Flowchart | √ |
|  |  | (b) Figure 1, Flowchart | √ |
|  |  | (c) Figure 1, Flowchart | √ |
| Descriptive data | 14* | (a) Give characteristics of study participants (eg demographic, clinical, social) and information on exposures and potential confounders – Page 7, heading baseline characteristics | √ |
|  |  | (b) Indicate number of participants with missing data for each variable of interest - NA | √ |
|  |  | (c) *Cohort study*—Summarise follow-up time (eg, average and total amount) – all patients had the same follow-up, 3 years | √ |
| Outcome data | 15* | *Cohort study*—Page 8, heading 3-year risk of type 2 diabetes | √ |
|  |  |  |  |
|  |  |  |  |
| Main results | 16 | (*a*) Page 8, heading 3-year risk of type 2 diabetes | √ |
|  |  | (*b*) Page 6, heading statistical analysis | √ |
|  |  | (*c*) Page 8, heading 3-year risk of type 2 diabetes | √ |
| Other analyses | 17 | Page 8, heading 3-sub-group analyses, page 10 younger patients, page 9 mortality | √ |
| Discussion | | |  |
| Key results | 18 | Page 9, paragraph 1 | √ |
| Limitations | 19 | Page 13, heading limitations | √ |
| Interpretation | 20 | Page 11, paragraph 2 | √ |
| Generalisability | 21 | Page 11, paragraph 2, page 10 paragraph 3 | √ |
| Other information | | |  |
| Funding | 22 | Provided in online form when article submitted | √ |

*Give information separately for cases and controls in case-control studies and, if applicable, for exposed and unexposed groups in cohort and cross-sectional studies.

**Note:** An Explanation and Elaboration article discusses each checklist item and gives methodological background and published examples of transparent reporting. The STROBE checklist is best used in conjunction with this article (freely available on the Web sites of PLoS Medicine at http://www.plosmedicine.org/, Annals of Internal Medicine at http://www.annals.org/, and Epidemiology at http://www.epidem.com/). Information on the STROBE Initiative is available at www.strobe-statement.org.
