# Supplementary material for: Stress Hyperglycaemia in Hospitalised Patients and Their 3-Year Risk of Diabetes: A Scottish Retrospective Cohort Study
Source: PLoS Med. 2014 Aug 19;11(8):e1001708. doi: 10.1371/journal.pmed.1001708 (PMC4138030; doi:10.1371/journal.pmed.1001708)
Supplement: Protocol S1 — Statistical analysis plan. (PDF) [file pmed.1001708.s010.pdf]

Statistical analysis plan

This file contains:-

1. Our protocol including planned statistical analyses as submitted to the Chief Scientist Office for Scotland (October 2012).
2. The final statistical analysis plan (July 2013).

RISK OF TYPE 2 DIABETES IN MIDDLE-AGED AND OLDER ADULTS FOLLOWING HYPERGLYCAEMIA DURING EMERGENCY HOSPITAL ADMISSION: A RETROSPECTIVE COHORT STUDY

1. INTRODUCTION

Hyperglycaemia in people without diabetes is common and is associated with adverse outcomes such as increased mortality and increased length of stay following admission with myocardial infarction, stroke, pneumonia and exacerbation of chronic obstructive pulmonary disease (COPD), as well as in general hospital admissions, particularly among the elderly.[1–8]

However, the longer term management of patients without diabetes who develop hyperglycaemia during acute illness is not well understood. Mechanisms implicated in precipitating hyperglycaemia of acute illness (stress hyperglycaemia) include release of counter-regulatory hormones such as glucagon, cortisol, catecholamine and growth hormone which oppose the effects of insulin and promote hepatic gluconeogenesis.[9] However, the extent to which this group of patients are predisposed to develop type2 diabetes is unknown.

The value of screening for type 2 diabetes in the general population remains controversial,[10] with an updated Health Technology Assessment expected later this year. Nevertheless, in the specific context of admission to hospital for acute coronary syndrome (ACS) the recent NICE guidelines recommend that patients with hyperglycaemia without diabetes should be informed that they have an increased risk of developing type 2 diabetes and should be screened for diabetes at least annually (http://www.nice.org.uk/guidance/CG130). However, this recommendation was necessarily made in the absence of data for the yield of screening as studies following-up patients with hyperglycaemia during ACS were small (a maximum of 630 patients with the majority having less than 200 patients) and of limited duration of follow-up (up to 3 months).[11–15]

Like acute coronary syndrome, chronic obstructive pulmonary disease (COPD) is one of the commonest causes of admission to hospital in Scotland (www.isdscotland.org/Health-Topics/Hospital-Care/Diagnoses/) and is the fourth leading cause of death worldwide (www.who.int/healthinfo/global_burden_disease/). Hyperglycaemia in patients hospitalised with exacerbation of COPD appears to be common (in one study 11% of patients had one or more venous glucose measurements >11.1mmol/l[5]). Hyperglycaemia has been associated with increased mortality and length of stay in patients admitted to hospital with COPD, and with intubation and death (as a composite end-point) in patients treated with non–invasive ventilation for exacerbation of COPD.[5,6] In addition to the mechanisms described above, corticosteroid prescription is common in exacerbation of COPD, and hyperglycaemia was found to be the most commonly reported side-effect of steroid treatment.[16] We did not find any studies that have reported the subsequent risk of developing diabetes in people admitted to hospital with COPD who had acute hyperglycaemia.

Several studies have examined the risk of subsequent diabetes among patients who had hyperglycaemia during hospital admission without selecting patient with specific diagnoses. Two were small convenience samples with less than 50% follow-up.[17,18] One study recruited 500 consecutive patients aged 40 years and older from an emergency department in the UK and identified that 36 patients without known diabetes had a capillary blood glucose of >7mmol/l of whom 13 subsequently met the criteria for type 2 diabetes on out-patient fasting blood glucose testing. However, follow-up was limited to one week, and the study lacked power to differentiate the risk according to age, sex or other characteristics.[19] Similarly, a retrospective cohort study of patients attending an emergency department in Northern England in 1994-1995 followed-up 317 patients with new hyperglycaemia (glucose >11.1 mmol/l) using a local diabetes register, GP records and hospital notes and found that approximately one third had been diagnosed with diabetes within 5 years.[20] However, this study was performed in a single centre, the

proportion of patients in whom glucose had been measured was not stated, and almost 30% of patients were lost to follow-up. Neither study followed-up patients with moderately raised venous glucose (6.1 mmol/l to 11.0 mmol/l).

Therefore, for emergency admissions in general, as well as for specific common conditions, the risk of subsequently developing type-2 diabetes by concentration of venous glucose during acute illness is unknown. Consequently, it is difficult for clinicians to adequately inform patients about their future risk, and to decide what level of follow-up testing is warranted. The information collected in this study will inform the debate about the value of screening for diabetes in high risk sub-groups.

We aim to help address this question using data collected routinely within the NHS in Scotland.

2. PILOT STUDIES

From an existing linked dataset (comprising admissions and diagnoses of diabetes from 2000 to 2007 for people included in the national population based register of people with diagnosed diabetes, the Scottish Care Information – Diabetes Collaboration dataset [SCI-DC]) we found that 1,185 Lothian and Fife residents aged 40 and older were diagnosed with type 2 diabetes between 1 month and 5 years of discharge from hospital in the financial year 2001/2002. There were 61,335 Fife and Lothian residents aged 40 and older with one or more emergency admissions that year (www.isdscotland.org/Health-Topics/Hospital-Care/Inpatient-and-Day-Case-Activity/).

We estimated the 5-year cumulative incidence following an emergency admission during the financial year 2001/02 from these numerator and denominator data. The prevalence of diabetes in the general population is approximately 5%.[21] Therefore, the five year cumulative incidence was 2.1%(95% CI 2.0 to 2.3) and 2.4% (95%CI 2.3 to 2.6) assuming that the prevalence of diabetes in hospitalised patients is 2 and 4 times that observed in the general population respectively.

Using data from other local cohorts we examined how frequently glucose was measured in two common causes of emergency admissions to hospital. Among patients with suspected acute coronary syndrome who had presented to hospital in South East Scotland, venous glucose was routinely measured 92% of patients (1402/1526) (unpublished data from GRACE study, personal communication Kathryn Caruthers). Similarly, in a case-series among 234 patients admitted to hospital with exacerbation of COPD in 4 hospitals across central Scotland, 207 patients did not have known previous diabetes. Among this group 197 (95%) had a venous admission glucose measured.[22]

Therefore, an appreciable number of people are diagnosed with type 2 diabetes within 5 years of discharge from hospital, and the majority of patients admitted with common emergencies have an admission venous glucose measured.

3. AIMS

The aim of this study is to estimate the 5-year incidence of type 2 diabetes among middle-aged and older people without diabetes following an emergency admission to hospital, and to describe how this incidence varies by the admission venous glucose level.

4. RESEARCH QUESTIONS

Among people without a diagnosis of diabetes on discharge from an emergency admission to hospital in Lothian and Fife and Greater Glasgow and Clyde 2004-2006:

1. What is the distribution of admission venous glucose concentrations among patients who had been admitted to hospital as an emergency?

2. What proportion of patients admitted to hospital as an emergency has follow-up venous glucose or HbA1c measured, what is the median time to measurement from discharge, and do these differ by admission glucose concentration?

3. What is the 5-year cumulative incidence (risk) of developing diabetes among patients who had been admitted to hospital as an emergency at different levels of admission venous glucose?

4. Do age, sex, deprivation (as measured by the Scottish Index of Multiple Deprivation), specialty (medicine and surgery) and pre-specified diagnoses of interest based upon International Classification of Diseases-coded definitions (including COPD, myocardial infarction, stroke and fracture) influence the outcomes identified in questions 1-3.

5. In exploratory analyses we will examine whether:-

- the risk of diabetes according to admission glucose differs by the presence/absence of pre-specified co-morbid conditions
- the risk of diabetes according to admission glucose differs by the presence/absence and/or level of C-reactive protein
- the risk of diabetes is most closely related to the first (admission), mean, peak or last glucose measure.

5. PLAN OF INVESTIGATION

Study design

We will employ a retrospective cohort design using data obtained from linking three databases. SCI-store (for laboratory test results), SCI-DC (for diagnosis of diabetes) and mortality linked acute hospital admission data (Scottish Morbidity Record, SMR01).

Study population

We will identify a cohort of all patients residing in Lothian and Fife and Greater Glasgow and Clyde Health boards aged 40 or older who had been discharged during calendar years 2004-2006 (inclusive) having been admitted as an emergency medical or surgical admission to a Lothian or Fife or Greater Glasgow and Clyde hospital from the SMR01 database.

Using the linked SCI-DC-SMR01 database, patients with prevalent diabetes (of any type) on or prior to the date of discharge will be excluded. Patients who are diagnosed with diabetes within 1 month of discharge will also be treated as prevalent cases in order to exclude patients who had been admitted to hospital primarily due to a complication of diabetes. Only the first eligible admission will be included in the analyses.

We will obtain information from the SCI-store database for this cohort on the first venous glucose measurement for the admission, as well as subsequent venous glucose or HbA1c measures following this initial glucose measure. We will also link these data to SCI-DC in order to obtain 5-year follow-up data (all years up to 2011) to identify incident diabetes. All three databases contain the unique Scotland-wide community health index (CHI) number which is a unique identifier which will allow deterministic linkage.

The risk of diabetes in patients discharged from hospital with no record of venous glucose measurement will be reported, but these patients will be excluded from other analyses.

Outcomes

Diabetes

Date of diagnosis of diabetes, and type of diabetes will both be defined using the most recent algorithm employed in SCI-DC which is derived from a number of items.[21] This definition will be used to identify both prevalent and incident type 2 diabetes.

Follow-up glucose or HbA1c measure

Any glucose or HbA1c measured after the discharge but not during any subsequent hospital admission will be defined as a follow-up glucose or HbA1c measure.

Exposure - Admission venous glucose

The first glucose measure during the admission will be defined as the admission glucose. We have not prospectively defined 'hyperglycaemia' as the aim of this study is to identify the risk of diabetes by different levels of venous glucose, but we will examine the effect of applying the standard international criteria for impaired glucose tolerance and diabetes.

Other predictors

Age at hospital discharge, sex and the Scottish Index of Multiple Deprivation (SIMD, an area based measure of socio-economic deprivation) will be obtained from the SMR-01 data.

Hospital admissions will be defined using continuous in-patient stays (CIS) which combine data from each event within hospital, which includes within hospital transfers. Diagnostic subgroups will be defined using the code recorded for the main diagnosis for the first event within a CIS. The tenth International Classification of Disease codes (ICD-10) codes will be used to define the specific conditions of interest (J40-44, I20-25, G45 & I60-67 and S02-S92 (code = Sx2, x ranges from 0 to 9) for COPD, MI, Stroke and fracture respectively) with other admissions being considered as a separate category.

Medical and surgical specialties will be defined according to administration codes used by ISD.

The major acute hospitals included in our analyses (Edinburgh Royal Infirmary, Western General Hospital Edinburgh, St John's Livingstone, Victoria Hospital Kirkcaldy and Queen Margaret Hospital Dunfermline) all performed above the Scottish average for coding of the main condition causing admission to hospital during the baseline period for our retrospective cohort study (www.isdscotland.org/Products-and-Services/Data-Quality/).

Statistical analyses

Initially, we will explore the association between subsequent risk of incident diabetes and admission venous glucose concentration, by the variables of interest by calculating and plotting the rates within appropriate categories (eg deciles and defined using standard criteria) of venous glucose.

We will report the proportion of patients who have a follow-up venous glucose or HbA1c measure and median time to measurement by admission venous glucose, age, sex and deprivation quintile.

We will calculate the predicted probability of developing incident diabetes in the 5 years following discharge (5-year cumulative incidence) using logistic regression models. We will present the overall probability, and the probabilities obtained from models adjusting for age, sex, SIMD quintile, and for particular specialties, as well as for the specific conditions identified on ICD code from the SMR-01 (see above).

We will examine whether the risk of subsequent diabetes is related (log) linearly to admission venous glucose using generalized additive models, graphically comparing any thresholds identified in our analyses to cut-offs currently used to screen for diabetes. We previously conducted a similar analysis (Figure 1) where we modelled the odds ratio for recurrent myocardial infarction or death by venous troponin concentration, comparing the identified threshold to existing cut-offs.

If strong associations are found, we will also explore whether admission venous glucose improves discrimination for risk of diabetes by estimating the area under the receiver operating characteristic curves.

In exploratory analyses, we will examine whether the association between diabetes and glucose during hospital admission is strongest for first (admission), mean, peak or last glucose measure.

We will also examine whether the predicted probability of developing incident diabetes in the 5 year following discharge differs by presence/absence of co-morbid conditions and c-reactive protein level at the time of discharge from hospital.

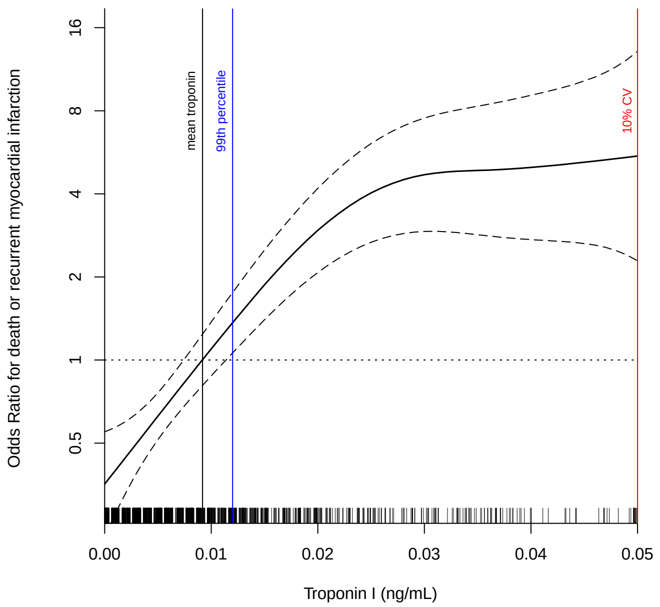

Figure 1 Odds ratio for recurrent myocardial infarction or death by troponin concentration among people presenting to hospital with chest pain- vertical lines indicate cut-offs currently used to diagnose myocardial infarction

Power calculation

Following comments at the initial review stage we have agreed to try and obtain similar data for Greater Glasgow and Clyde (note subject to agreement at these sites). However, these power calculations are based upon Lothian and Fife numbers.

Using data from SCI-DC for Lothian and Fife, over a three-year period (financial years 2000/1 to 2002/03) 3,028 patients were diagnosed with type-2 diabetes between 1 month and 5 years after hospital discharge following an emergency medical or surgical admission. From nationally published data (www.isdscotland.org/Health-Topics/Hospital-Care/Inpatient-and-Day-Case-Activity/) for the same geography a total of 181,103 patients aged 40 or older had emergency admissions. Using conservative assumptions that 80% of all patients did not have prevalent diabetes, 20% of people were double-counted from having been admitted in more than one financial year, and admission venous glucose was not measured in 20%, we estimate that the number of patients without known diabetes discharged from hospital as an emergency with glucose measured during this time period would be approximately 92,725.

Consequently, we would have a 90% power to detect a difference of 0.8% in the cumulative incidence of diabetes in the top decile (n = 9,273) of admission venous glucose (relative to another decile n = 9,273) at the 0.05 significance level, assuming the risk in the comparator decile was 2.6% (risk obtained from the estimates presented in pilot studies).

Similarly for precision, the conservative approach of treating each decile of admission plasma glucose as a discrete category would provide 95% confidence intervals around the estimated 5 year cumulative incidence for each decile of +/- 0.4% for a cumulative incidence of 3% to +/- 0.6% for a cumulative incidence of 9%.

Of the 3,028 patients who developed incident diabetes 108 were discharged following a diagnosis of COPD, 495 with myocardial infarction, 247 with stroke and 250 with fracture (ICD codes J40-44, I20-25, G45- I60-67 and S02-S92 respectively). For the first admission during this time period the three commonest specialties were General Medicine, General Surgery and Trauma and Orthopaedic surgery (n=1597, n=517 and n=204 respectively). Consequently, we will be able to explore the five-year risk of

diabetes in these different sub-groups, albeit with wider confidence intervals around the estimates of incidence.

Both emergency hospital admissions and the incidence of diabetes have increased over the past decade, and therefore both the numerator and denominator are likely to be higher in the 2004 to 2006 cohort we will include than in the 2000 to 2003 cohort we used for these power calculations.

Data processing and analysis

Details of the statistical analyses are given under study design.

We will adopt a similar procedures to those used in previous similar studies using linked data conducted within our department having obtained appropriate ethical, Caldicott and Privacy Advisory Committee approvals. SCI-stores for Fife and Lothian and Greater Glasgow and Clyde are held within NHS Fife and NHS Lothian and Greater Glasgow and Clyde respectively, and SCI-DC and SMR01 are stored within ISD. We will securely transfer data from SCI-store from each health board area to ISD. ISD will link the data within a safe haven. Identification of conditions and specialties of interest from ICD and administrative codes will also be performed by ISD. Anonymised data will be provided via a secure mechanism and stored on a secure, encrypted password protected server within University of Edinburgh, to which access will be limited to named project staff.

Analyses will be conducted within the University network by experienced statistician Cat Graham. David McAllister, Sarah Wild, Katherine Hughes, Nazir Lone and John McKnight will provide clinical and epidemiological support for the study.

12. REFERENCES

1. Capes SE, Hunt D, Malmberg K, Gerstein HC. Stress hyperglycaemia and increased risk of death after myocardial infarction in patients with and without diabetes: a systematic overview. Lancet. 2000 Mar 4;355(9206):773–8.

2. Kosiborod M, Rathore SS, Inzucchi SE, Masoudi FA, Wang Y, Havranek EP, et al. Admission glucose and mortality in elderly patients hospitalized with acute myocardial infarction: implications for patients with and without recognized diabetes. Circulation. 2005 Jun 14;111(23):3078–86.

3. Capes SE, Hunt D, Malmberg K, Pathak P, Gerstein HC. Stress hyperglycemia and prognosis of stroke in nondiabetic and diabetic patients: a systematic overview. Stroke. 2001 Oct;32(10):2426–32.

4. McAlister FA, Majumdar SR, Blitz S, Rowe BH, Romney J, Marrie TJ. The relation between hyperglycemia and outcomes in 2,471 patients admitted to the hospital with community-acquired pneumonia. Diabetes Care. 2005 Apr;28(4):810–5.

5. Baker EH, Janaway CH, Philips BJ, Brennan AL, Baines DL, Wood DM, et al. Hyperglycaemia is associated with poor outcomes in patients admitted to hospital with acute exacerbations of chronic obstructive pulmonary disease. Thorax. 2006 Apr 1;61(4):284–9.

6. Chakrabarti B, Angus RM, Agarwal S, Lane S, Calverley PMA. Hyperglycaemia as a predictor of outcome during non-invasive ventilation in decompensated COPD. Thorax. 2009 Oct;64(10):857–62.

7. Sleiman I, Morandi A, Sabatini T, Ranhoff A, Ricci A, Rozzini R, et al. Hyperglycemia as a predictor of in-hospital mortality in elderly patients without diabetes mellitus admitted to a sub-intensive care unit. J Am Geriatr Soc. 2008 Jun;56(6):1106–10.

8. Umpierrez GE, Isaacs SD, Bazargan N, You X, Thaler LM, Kitabchi AE. Hyperglycemia: an independent marker of in-hospital mortality in patients with undiagnosed diabetes. J. Clin. Endocrinol. Metab. 2002 Mar;87(3):978–82.

9. Dungan KM, Braithwaite SS, Preiser J-C. Stress hyperglycaemia. Lancet. 2009 May 23;373(9677):1798–807.

10. Waugh N, Scotland G, McNamee P, Gillett M, Brennan A, Goyder E, et al. Screening for type 2 diabetes: literature review and economic modelling. Health Technol Assess. 2007 May;11(17):iii-iv, ix-xi, 1–125.

11. Ishihara M, Inoue I, Kawagoe T, Shimatani Y, Kurisu S, Hata T, et al. Is admission hyperglycaemia in non-diabetic patients with acute myocardial infarction a surrogate for previously undiagnosed abnormal glucose tolerance? Eur. Heart J. 2006 Oct;27(20):2413–9.

12. Okosieme OE, Peter R, Usman M, Bolusani H, Suruliram P, George L, et al. Can admission and fasting glucose reliably identify undiagnosed diabetes in patients with acute coronary syndrome? Diabetes Care. 2008 Oct;31(10):1955–9.

13. Norhammar A, Tenerz A, Nilsson G, Hamsten A, Efendíc S, Rydén L, et al. Glucose metabolism in patients with acute myocardial infarction and no previous diagnosis of diabetes mellitus: a prospective study. Lancet. 2002 Jun 22;359(9324):2140–4.

14. Tenerz A, Norhammar A, Silveira A, Hamsten A, Nilsson G, Rydén L, et al. Diabetes, insulin resistance, and the metabolic syndrome in patients with acute myocardial infarction without previously known diabetes. Diabetes Care. 2003 Oct;26(10):2770–6.

15. Oswald GA, Yudkin JS. Hyperglycaemia following acute myocardial infarction: the contribution of undiagnosed diabetes. Diabet. Med. 1987 Feb;4(1):68–70.

16. Wood-Baker RR; Gibson PG; Hannay M; Walters EH; Walters JAE, The Cochrane Collaboration Web Team. Systemic corticosteroids for acute exacerbations of chronic obstructive pulmonary disease [Internet]. [cited 2008 Dec 24]. Available from: http://www.cochrane.org/reviews/en/ab001288.html

17. Silverman RA, Thakker U, Ellman T, Wong I, Smith K, Ito K, et al. Hemoglobin A1c as a screen for previously undiagnosed prediabetes and diabetes in an acute-care setting. Diabetes Care. 2011 Sep;34(9):1908–12.

18. Charfen MA, Ipp E, Kaji AH, Saleh T, Qazi MF, Lewis RJ. Detection of undiagnosed diabetes and prediabetic states in high-risk emergency department patients. Acad Emerg Med. 2009 May;16(5):394–402.

19. George PM, Valabhji J, Dawood M, Henry JA. Screening for Type 2 diabetes in the accident and emergency department. Diabet. Med. 2005 Dec;22(12):1766–9.

20. Wahid ST, Sultan J, Handley G, Saeed BO, Weaver JU, Robinson ACJ. Serum fructosamine as a marker of 5-year risk of developing diabetes mellitus in patients exhibiting stress hyperglycaemia. Diabet. Med. 2002 Jul;19(7):543–8.

21. Scottish Diabetes Survey Monitoring Group. Scottish Diabetes Survey 2010 [Internet]. Diabetes in Scotland. [cited 2012 Mar 8]. Available from: http://www.diabetesinscotland.org.uk/Publications.aspx

22. McAllister DA, Maclay JD, Mills NL, Leitch A, Reid P, Carruthers R, et al. Diagnosis of myocardial infarction following hospitalisation for exacerbation of COPD. ERJ(epub ahead of print). 2012 Feb 9;

STATISTICAL ANALYSIS PLAN

Aim of Study

- The aim of this study is to estimate the 3-year incidence of type 2 diabetes among middle-aged and older people without diabetes following an emergency admission to hospital.
- Describe how this incidence rate varies by the admission venous glucose level.

Research Questions

Among people without a diagnosis of diabetes on discharge from an emergency admission to hospital in Lothian and Fife, and Greater Glasgow and Clyde 2004-2008:

1. What is the distribution of admission venous glucose concentrations among patients who had been admitted to hospital as an emergency?
2. What proportion of patients admitted to hospital as an emergency have a follow-up venous glucose or HbA1c measured? What is the median time to measurement from discharge? Do these differ by admission glucose concentration?
3. What is the 3-year cumulative incidence (risk) of developing diabetes among patients who had been admitted to hospital as an emergency at different levels of admission venous glucose?
4. Does age, sex, deprivation, speciality and pre-specified diagnoses of interest based upon International Classification of Diseases-coded definitions (including COPD, myocardial infarction, stroke and fracture) influence the outcomes identified in questions 1-3.
5. In an exploratory analyses we will examine whether:
    - The risk of diabetes according to admission glucose differs by the presence/absence of pre-specified co-morbid conditions.
    - The risk of diabetes according to admission glucose differs among patients without elevated WCC.

Dataset

The dataset shall only include patients who:

The University of Edinburgh is a charitable body, registered in Scotland, with registration number SC005336.

- are aged 40 years or over at the time of discharge.
- were an emergency admission (definition includes WCC measured)
- do not have prevalent diabetes (defined as diabetes on or prior to 30 days after the discharge from hospital).

Analysis
0a proportion with glucose measured by age, sex, simd, specialty, condition, hospital.
0b proportion who develop diabetes within 3 years with and without glucose measured

Further analyses (*unless specified*) shall only include patients who have had a glucose measurement taken within 2 days of admission.

1. A demographic statistics table shall present patient characteristics for the following shall be presented:

  - Age
  - Sex
  - SIMD
  - White blood Cell Count (WCC) – median, IQ range
  - C-Reactive Protein (CRP) – median, IQ range
  - Glucose (i.e. categorical) – less than 4, 4 to 6.9, 7 to 11, 11.1 to 15, 15.1 to 20, more than 20
  - Glucose (median IQR) may as well have both
  - Co-morbid conditions (i.e. Charlson) – 0, 1-2, 3-4, 5-6 >6
  - Speciality subgroups (i.e. medical, surgical, ICU)
  - Admissions to hospital because of one of the diagnosis subgroups:
    - Myocardial infarction
    - Stroke
    - Fracture
    - Exacerbation of chronic obstructive pulmonary disease (COPD)
  - Incident diabetes (i.e. yes/no)
  - Days from admission to glucose measurement
  - Categorical, WCC >11

The University of Edinburgh is a charitable body, registered in Scotland, with registration number SC005336.

2. A set of cumulative distribution plots of admission glucose levels shall be presented for "all" patients and separately for age groups, sex, specialty and diagnosis subgroups.

3. From the data, the proportion of patients who have follow-up venous glucose and HbA1c measures within various times of discharge shall be presented. For follow-up venous glucose the number and proportion of follow-up glucose measures according to the cut-offs will also be presented (less than 4, 4 to 6.9, 7 to 11, 11.1 to 15, 15.1 to 20, more than 20).Descriptive statistics of these results shall also be produced for "all" patients with separate tables for medical, surgical and diagnosis subgroups.

4. Logistic regression shall be used to estimate the 3 year risk of diabetes according to admission venous glucose concentration for "all" patients stratified by age, sex, SIMD quintile, elevated WCC (WCC >11 = "elevated WCC".

5. For each diagnosis subgroup separately, logistic regression shall be used to estimate the 3 year risk of diabetes according to admission venous glucose concentration.

6. Logistic regression shall be used to estimate the 3 year risk of diabetes according to admission venous glucose concentration in patients by speciality (medical, surgical) in separate models. Where a patient has both medical and surgical specialities it shall be the first speciality that will be used (specified in the data).
RESULTS OF ANALYSES 3 TO 6 WILL BE PRESENTED GRAPHICALLY AND IN TABLES.

7. The cumulative incidence of diabetes by 3 year follow-up time[1] (with confidence intervals) according to admission glucose level shall be presented for "all" patients. A cumulative incidence plot shall also be presented.

8. The cumulative incidence of diabetes by admission glucose levels (with confidence intervals) shall be presented and stratified by age groups, sex, speciality and diagnosis subgroups. Sets of cumulative incidence plots stratified by the different subgroups shall also be presented.

[1] From discharge to diagnosis

The University of Edinburgh is a charitable body, registered in Scotland, with registration number SC005336.

9a. Explore whether admission glucose measurement can be used to discriminate the 3 year risk of diabetes using ROC analysis and estimating the area under the ROC curve.

9b. Only 22% of patients had more than one in-patient glucose measure. Therefore, sensitivity analyses using mean, peak glucose measurement will not be performed.

10. Logistic regression shall be used to examine if the 3 year risk of diabetes can be predicted using admission glucose concentration stratified by co-morbidity (Charlson index 0, 1 to 4, 5 to 6 and >6), elevated WCC (WCC >11).

The University of Edinburgh is a charitable body, registered in Scotland, with registration number SC005336.
